# DSNetwork: An Integrative Approach to Visualize Predictions of Variants’ Deleteriousness

**DOI:** 10.3389/fgene.2019.01349

**Published:** 2020-01-17

**Authors:** Audrey Lemaçon, Marie-Pier Scott-Boyer, Régis Ongaro-Carcy, Penny Soucy, Jacques Simard, Arnaud Droit

**Affiliations:** Genomics Center, Centre Hospitalier Universitaire de Quebec—Université Laval Research Center, Quebec, QC, Canada

**Keywords:** fine-mapping analysis, variant prioritization, decision support, deleteriousness prediction, network visualization

## Abstract

One of the most challenging tasks of the post-genome-wide association studies (GWAS) research era is the identification of functional variants among those associated with a trait for an observed GWAS signal. Several methods have been developed to evaluate the potential functional implications of genetic variants. Each of these tools has its own scoring system, which forces users to become acquainted with each approach to interpret their results. From an awareness of the amount of work needed to analyze and integrate results for a single locus, we proposed a flexible and versatile approach designed to help the prioritization of variants by aggregating the predictions of their potential functional implications. This approach has been made available through a graphical user interface called DSNetwork, which acts as a single point of entry to almost 60 reference predictors for both coding and non-coding variants and displays predictions in an easy-to-interpret visualization. We confirmed the usefulness of our methodology by successfully identifying functional variants in four breast cancer and nine schizophrenia susceptibility loci.

## Introduction

Since 2006, thousands of susceptibility loci have been identified through Genome-Wide Association Studies (GWAS) for numerous traits and complex diseases, including breast cancer (MacArthur et al., 2017). GWAS build on the concept of linkage disequilibrium (LD) to identify statistical associations between genetic variants and diseases (Visscher et al., 2017). While this approach is powerful for locus discovery, it cannot distinguish between truly causal variants and non-functional highly correlated neighboring variants. Thus, for the vast majority of these loci, the causal variant(s) and their functional mechanisms have not yet been elucidated.

Statistical fine-mapping analyses combined with the functional annotation of genetic variants can help pinpoint the genetic variant (or variants) responsible for complex traits, or at least narrow down the number of variants underlying the observed association for further functional studies. In this regard, tremendous efforts have been put forth to assist the functional assessment of variants at risk loci and numerous scoring methods and tools have been developed to predict the deleteriousness of variants based on a number of characteristics such as sequence conservation, characteristics of amino acid substitution, and location of the variant within protein domains or three-dimensional protein structure.

In recent years, efforts have been made towards the aggregation of many different functional annotations resulting from these scoring methods in a single integrative value called metascore (Ionita-Laza et al., 2016; Feng, 2017), an approach that seems to yield better performances than any predictor individually (Dong et al., 2015). Although these methods demonstrate themselves to be useful, they have some limitations, notably not being directly comparable to one another due to integration of different sets of annotations or different weighting of these annotations, and sometimes having contradictory results.

In order to allow a quick survey of a wide range of predictors for a given list of variants and assist in the interpretation of the resulting prediction scores, we propose a flexible and integrative method capable of gathering information from multiple sources in an easy-to-interpret representation rather than a static new metascore. For this purpose, we created a single point of entry fetching predictors for coding and non-coding variants and presenting them as a network, where the nodes illustrate the scores of each predictor for a given variant and the edges the LD between variants. The network is built with the aim of rendering the predictor results easier to peruse during analyses involving multiple variants, and therefore, assist in the variant prioritization process in the context of fine-mapping analyses.

This approach has been made available through a graphical user interface (GUI) stand-alone application called DSNetwork. The tool is freely available *via* bitbucket repository and is also accessible through our portal for demonstration purpose at: http://romix.genome.ulaval.ca/dsnetwork/.

## Materials and Methods

### Annotations Retrieval

Variant annotations and scoring data are fetched on-the-fly from MyVariant.info high-performance web services (Xin et al., 2016) using their third-party R package. SNPnexus (Dayem Ullah et al., 2018) scorings are fetched upon request through a Python script kindly provided by the SNPnexus team. Due to their novelty and relevance for our purpose, three complementary whole genome resources are included: LINSIGHT (Huang et al., 2017), BayesDel (Feng, 2017), and predictions and sequence constraint data (di Iulio et al., 2018), which can be used as a proxy to score functionality and the consequences of mutations. BayesDel, LINSIGHT, and Context-Dependent Tolerance scores were extracted from a local copy. A description of the integrated predictors is available in the **Supplementary Material**.

LD data are computed from 1000 Genomes Phase 3 (1000 Genomes Project Consortium et al., 2015).

### Visual Integration

Prediction result for variants of interest are displayed as a network, whose components, namely, the edges and nodes, are used to convey different types of information in an easy-to-comprehend way.

The following paragraphs describe DSNetwork’s approach through the hypothetical analysis of a loci containing five variants rs4233486, rs35054111, rs11808410, rs11804913, and rs7554973 using the deleteriousness scores of five distinct fictive predictors A, B, C, D, and E. **Table 1** summarizes the scores generated by these five predictors, reflecting their predictions regarding the functional impacts of the candidate variants.

**Table 1:** Deleterious scores generated by five different approaches.

	A	B	C	D	E
rs4233486	0.13	0.4	0.78	0.23	0.12
rs35054111	NA	0.7	0.21	NA	0.43
rs11808410	0.51	0.4	0.21	0.2	0.77
rs11804913	0.01	0.4	0.21	0.3	0.37
rs7554973	0.2	0.5	0.55	NA	0.01

**Figure 1:**
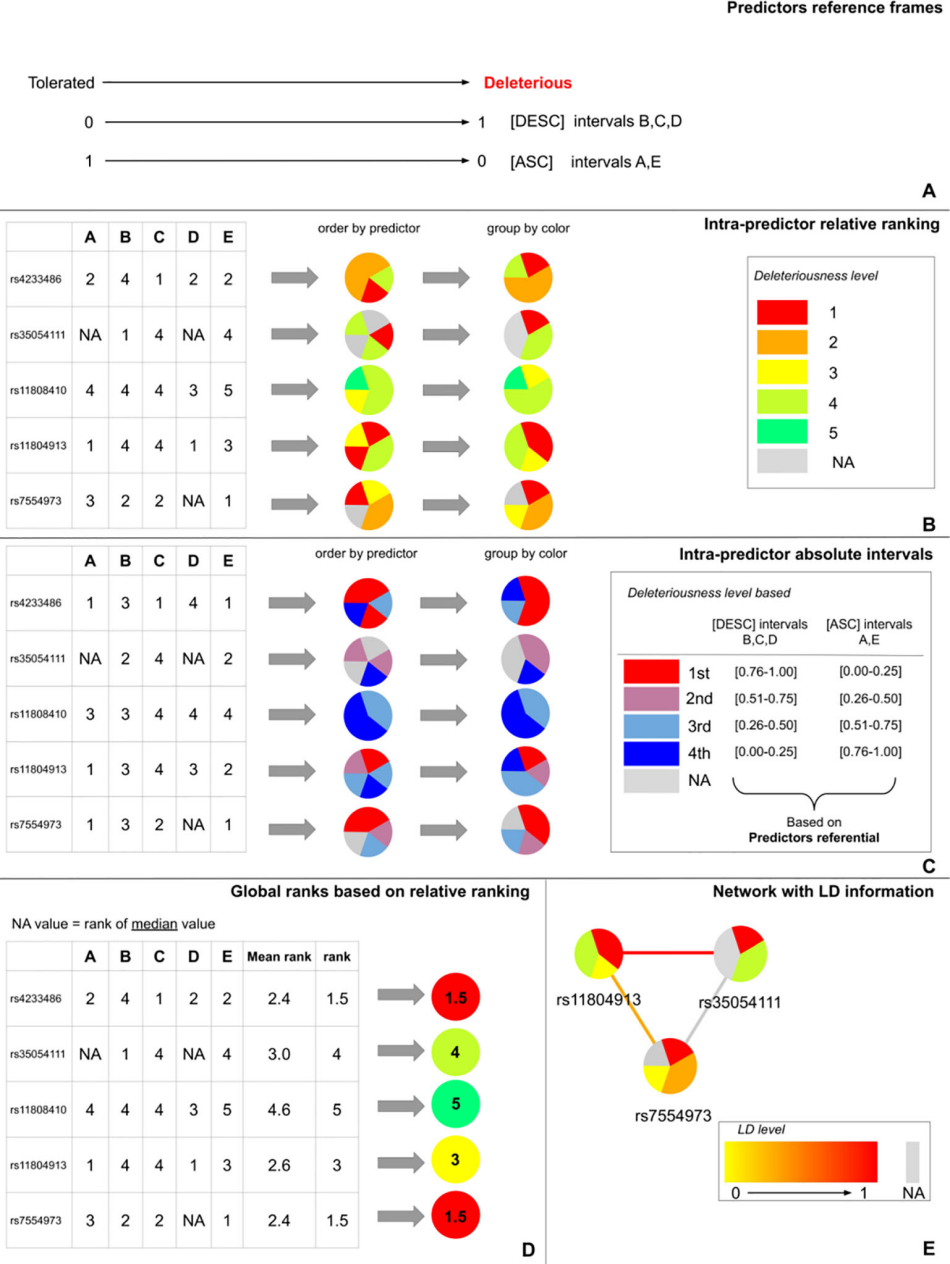
DSNetwork visual approach. **(A)** Representation of predictors reference frames illustrating each approach boundaries and direction. **(B)** Representation of intra-predictors ranking based on the predictors reference frame. **(C)** Representation of intra-predictors absolute score intervals based on the predictors reference frame. **(D)** Representation of the global mean rank. **(E)** The edges between the nodes can be used to map Linkage Disequilibrium (LD) levels between two variants.

DSNetwork integrates the characteristics of the different predictors and creates a reference frame containing the lower and upper boundaries as well as the direction [ascending (ASC) or descending (DESC)] of their prediction scores (**Figure 1A**). The direction is used to rank variants from the most deleterious to the least deleterious on the basis of their respective scores. The boundaries are used to establish the absolute deleteriousness level of each variant. Once the different reference frames are integrated, they can be used to prioritize the variants according to three types of representations: the intra-predictor relative ranks, the intra-predictor absolute scores, and the global ranks.

#### Intra-Predictor Ranks

Intra-predictor ranks allow the prioritization of a list of variants relative to one another. According to the reference frames illustrated in **Figure 1A**, the five predictors produce scores ranging from 0 to 1. We can classify the five variants of interest from the most deleterious (rank 1) to the least deleterious (rank 5) with each predictor. In order to summarize this information in an easy-to-interpret representation, each variant is depicted as a pie chart where each slice represents the rank of the variant for one of the predictors. Thus, in the current analysis, five pie charts are generated and each pie chart is divided by five slices of the same size. We used a color gradient ranging from red to green, where red corresponds to the most deleterious variant (rank 1) among the candidates for a given predictor. The gray color represents missing data. **Figure 1B** depicts the pie charts generated for the five candidate variants. The slices can be ordered by color to allow easy identification of variants that appear the most deleterious across predictors.

#### Intra-Predictor Absolute Scores

Intra-predictor absolute scores allow prediction of variant deleteriousness in reference to the thresholds established for a particular predictor. Given these boundaries, we can determine where each variant is located on the deleteriousness spectrum for each predictor. We chose to divide the score range of each approach into 20 equal intervals. This number of intervals was chosen as a compromise between granularity and readability. The first interval contains the most deleterious scores and the 20th, the least deleterious. Thus, the annotation scores obtained for each variant are translated into their corresponding intervals. This allows the user to know if a variant is predicted as deleterious by a particular approach without having to know the implementation details of this approach. For clarity purposes, in this example the range of scores has been divided into four intervals (instead of 20) (**Figure 1C**).

As for intra-predictor ranks, each variant is depicted as a pie chart where each slice represents the score interval of the variant for a particular predictor. We used a color gradient ranging from red to blue. The red color represents the most deleterious interval for a given predictor. The gray color represents missing data. **Figure 1C** depicts the pie charts generated for the five candidate variants. The slices can be ordered by color to easily identify variants with the most predictions of deleteriousness.

#### Global Ranking

In order to further facilitate the prioritization, we propose to summarize the information regarding the relative ranks in an overall rank for each variant. To do so, we calculate the average rank of each variant based on its intra-predictor ranks. Then, we order the variants according to their average rank. Variants with the lowest average ranks are considered as the best candidates for being deleterious. Because in some cases there may be missing values for some of the predictors when analyzing a specific set of variants, we propose three strategies for calculating a consistent average rank, which will be comparable between variants and which will take into account these missing values: 1) replace missing values with the median value (default one); 2) replace missing values with the average value; or 3) systematically attribute missing values the “worst” rank. Once the necessary substitutions are made, the average ranks can be calculated and the global ranks generated. As for the intra-predictor scores and ranks, the global ranks are made available for each variant under the form of a pie chart where the rank is represented by a color gradient ranging from red to green. The color red represents the most deleterious variant among the candidates for all approaches (**Figure 1D**).

#### Variants Network

DSNetwork offers the possibility to simply visualize scores and LD between variants in order to identify potential haplotypes through an interactive interface. Users can interact with the network using the mouse by scrolling in and out to zoom, or double-click on a variant node to display variant annotation details among other features. They can also update the predictors used to prioritize the variants. As displayed in **Figure 1E**, edges between nodes can be used to map LD levels between two variants. LD (squared correlation *r* ^2^) is based on a user-chosen 1000 genomes population and is represented by an absolute color gradient ranging from yellow to red. Red indicates a high disequilibrium. The gray color represents the missing information. By default, no LD data are shown. To map LD on the network edges, users have to choose a population from 1000 Genomes and can restrict the LD range to display for a particular variant.

### Implementation

**Figure 2:**
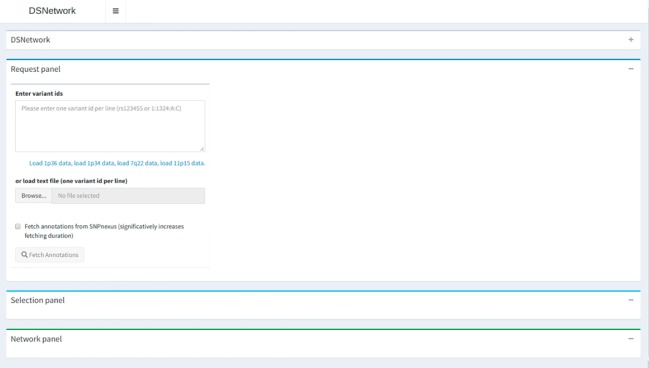
Architecture overview. The first section is dedicated to user input and parameters for data retrieval. The middle panel presents a relevant subset of annotations for each submitted variant and enables the selection of variants to be integrated in the final visualization. The bottom part on the interface is dedicated to the integrated visualization of the deleteriousness predictions displayed as a network.

DSNetwork was created using the Shiny framework (Chang et al., 2017). This tool provides users with deleteriousness predictions for a selected set of coding and non-coding human Single Nucleotide Variants (SNVs) and short inserts and deletions (InDels) (hg19 build) and generates a set of prioritized results for further analysis. These prediction scores are recovered from several trusted sources and presented in a cross-platform, user-friendly web interface. The interface is organized in three sections, namely, Input, Selection, and Visualization, as illustrated and described in **Figure 2**. For complete usage guide, see the **Supplementary Material**. DSNetwork is encapsulated using Docker platform to guarantee the cross-platform compatibility. The source code and installation procedure are available at https://bitbucket.org/vmtrap/dsnetwork_deploy/src/master/. The tool can be installed on all operating systems supporting Docker Engine (see supported platforms at https://docs.docker.com/install/) and is also accessible through our portal for demonstration purpose at: http://romix.genome.ulaval.ca/dsnetwork/.

### Case Studies

We chose to demonstrate the utility of DSNetwork in the context of the functional analysis of four breast cancer susceptibility loci identified through the latest published breast cancer association study (full description in Michailidou et al., 2017) and nine loci reported in the latest published study on schizophrenia susceptibility (full description in Huo et al., 2019). Michailidou et al. (2017) report the discovery of 65 new breast cancer risk loci and deepens the functional characterization for four regions, namely, 1p36, 1p34, 7q22, and 11p15. For each of these regions, the authors defined sets of credible risk variants (CRVs) and investigated their impact through functional assays in order to identify the functional variants. Huo et al. (2019) investigated over 180 loci reported to be associated with schizophrenia in several GWA studies and prioritized regulatory single-nucleotide polymorphisms (SNPs) at these risk loci. They deepen the functional validation of 10 variants from nine different loci.

## Results and Discussion

### Prioritization of Four Breast Cancer Susceptibility Loci

The original study by Michailidou et al. (2017) reported 65 novel breast cancer susceptibility loci. For each of these regions, they defined a set of CRV containing variants with P-values within two orders of magnitude of the most significant SNPs in this region. They then selected four loci for further evaluation, namely, 1p36, 1p34, 7q22, and 11p15. Initially, these four regions contained, respectively, 54, 13, 19, and 85 significantly associated variants. The p-value cutoff enabled them to reduce the number of variants to, respectively, 1, 4, 6, and 19 CRVs. The list of variants for these loci was extracted from the original paper’s Supplementary Tables 8 and 13 in the context of the current analysis. Following data extraction, the analysis procedure was: 1) upload the variants of interest on the web tool, 2) fetch the annotations, 3) visualize the variants through the overview plot, 4) visualize the available deleteriousness scores through the relative ranking in the decision network, 5) use absolute interval visualizations to identify the best candidates, and finally 6) conclude.

#### Locus 1p36

This region contains a single CRV, rs2992756 (P = 1.6×10^−15^). For demonstration purposes, we selected the 30 most associated variants in this region to put to the test. Among these 30 variants, 2 variants (rs200439143, rs71018084) weren’t annotated by DSNetwork because of their absence from MyVariant.info service, and 24 were identified as regulatory variants and 4 as non-synonymous variants. For the purposes of our analysis, we focused on the regulatory variants.

**Figure 3:**
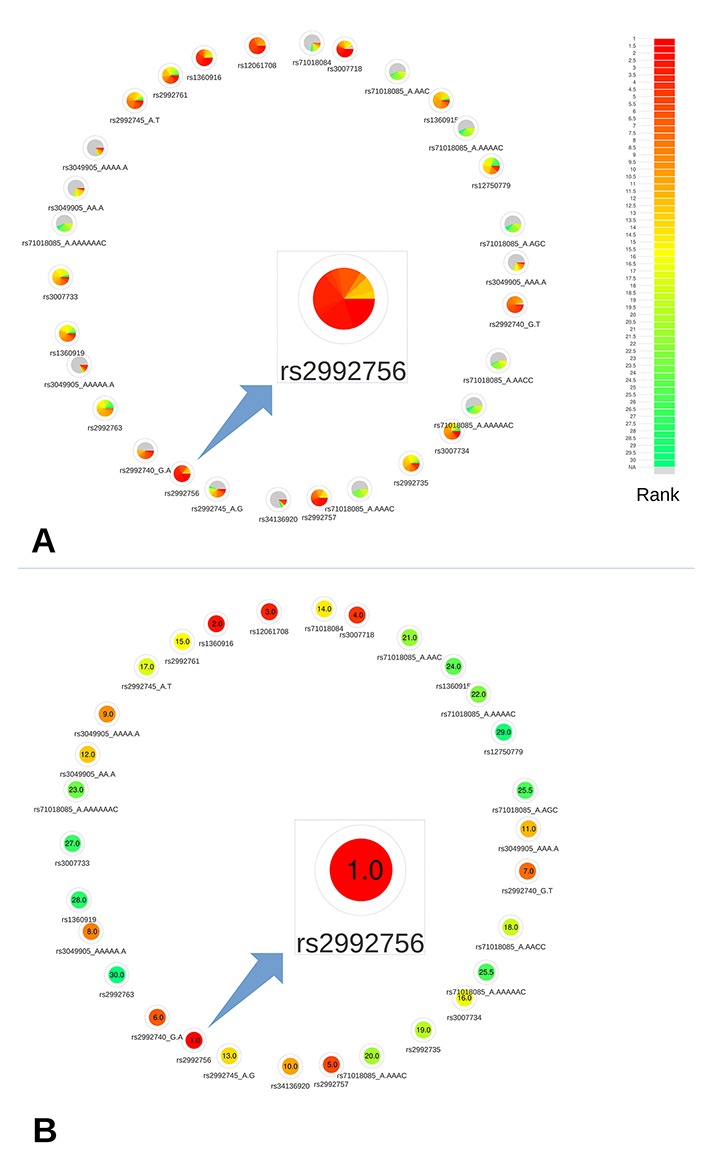
Networks representing the 30 most significant variants associated with breast cancer at the 1p36 locus. **(A)** All available predictions represented under the form of relative rank grouped by color. **(B)** Global ranking representing the mean relative ranks with missing values substituted by the median value. Based on the deleteriousness scores available for this subset of variants, a quick overview of variant nodes has allowed to easily identify rs2992756 as the best candidate.

Based on the deleteriousness scores available for this subset of variants, a quick overview of variant nodes has allowed to easily identify rs2992756 as the best candidate. Indeed, the node for this variant contained the largest proportion of red, indicating a high ranking for most of the scoring approaches (**Figure 3A**). To confirm this observation, we used the relative rank visualization (**Figure 3B**). The mean rankings of variants, clearly materialized by both the color code and the values, enabled the confirmation of rs2992756 as the best candidate among the 30 most breast cancer-associated variants at the 1p36 locus. Using reporter assays, Michailidou et al. (2017) demonstrated that the presence of the risk T-allele of this variant within *KLHDC7A* promoter significantly lowers its activity.

#### Locus 1p34

This region contains four CRVs among 13 significantly associated variants. All the variants were found by DSNetwork and identified as regulatory variants.

**Figure 4:**
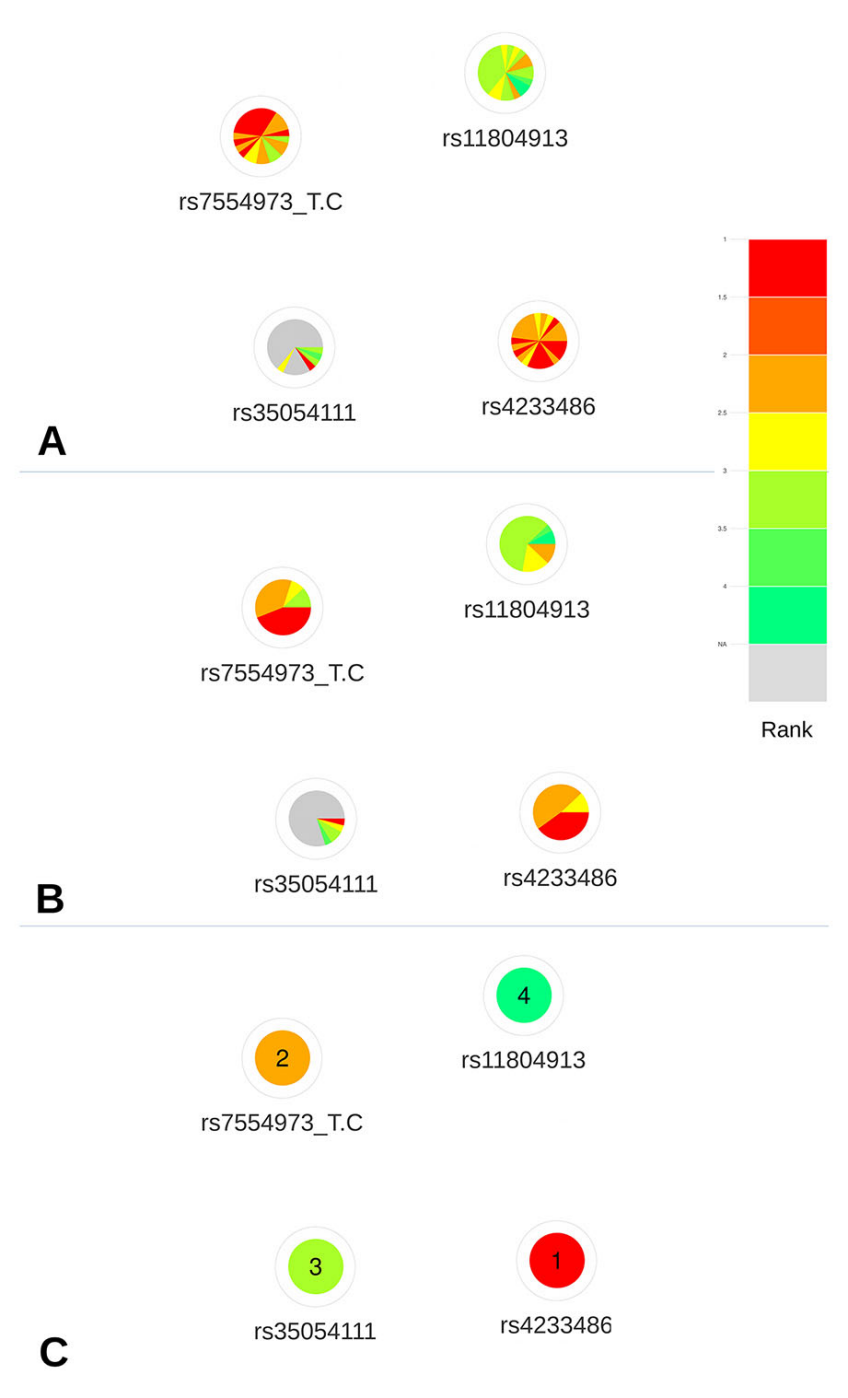
Networks representing the four CRVs associated variants with breast cancer at the 1p34 locus. **(A)** All available predictions represented under the form of relative rank ordered by predictors. **(B)** All available predictions represented under the form of relative rank grouped by color. **(C)** Global ranking representing the mean relative ranks with missing values substituted by the median value. Based on the deleteriousness scores available for this subset of variants, a quick overview of variant nodes has allowed to easily identify two variants, rs42334486 and rs7554973, as the best candidates.

Based on the deleteriousness scores available for this subset of variants, a quick overview of variant nodes has allowed to easily identify two variants, rs42334486 and rs7554973, as the best candidates. Indeed, the nodes for these variants contained the largest proportion of red and orange indicating a good ranking of these variants for most of the scoring approaches (**Figure 4A**). The sorting by color (**Figure 4B**) facilitated the prioritization of these two variants, which initially seemed to present the same proportion of high ranks. The visualization of the mean ranking confirms rs4233486 as the most credible candidate among the CRVs (**Figure 4C**). This observation is in accordance with results from Michailidou et al. (2017), which demonstrated, using reporter assays, that the presence of the risk T-allele of this variant within a putative regulatory element (PRE) reduced *CITED4* promoter activity.

#### Locus 7q22

This region contains six CRVs among 19 significantly associated variants. All the variants were found by DSNetwork and identified as regulatory variants.

**Figure 5:**
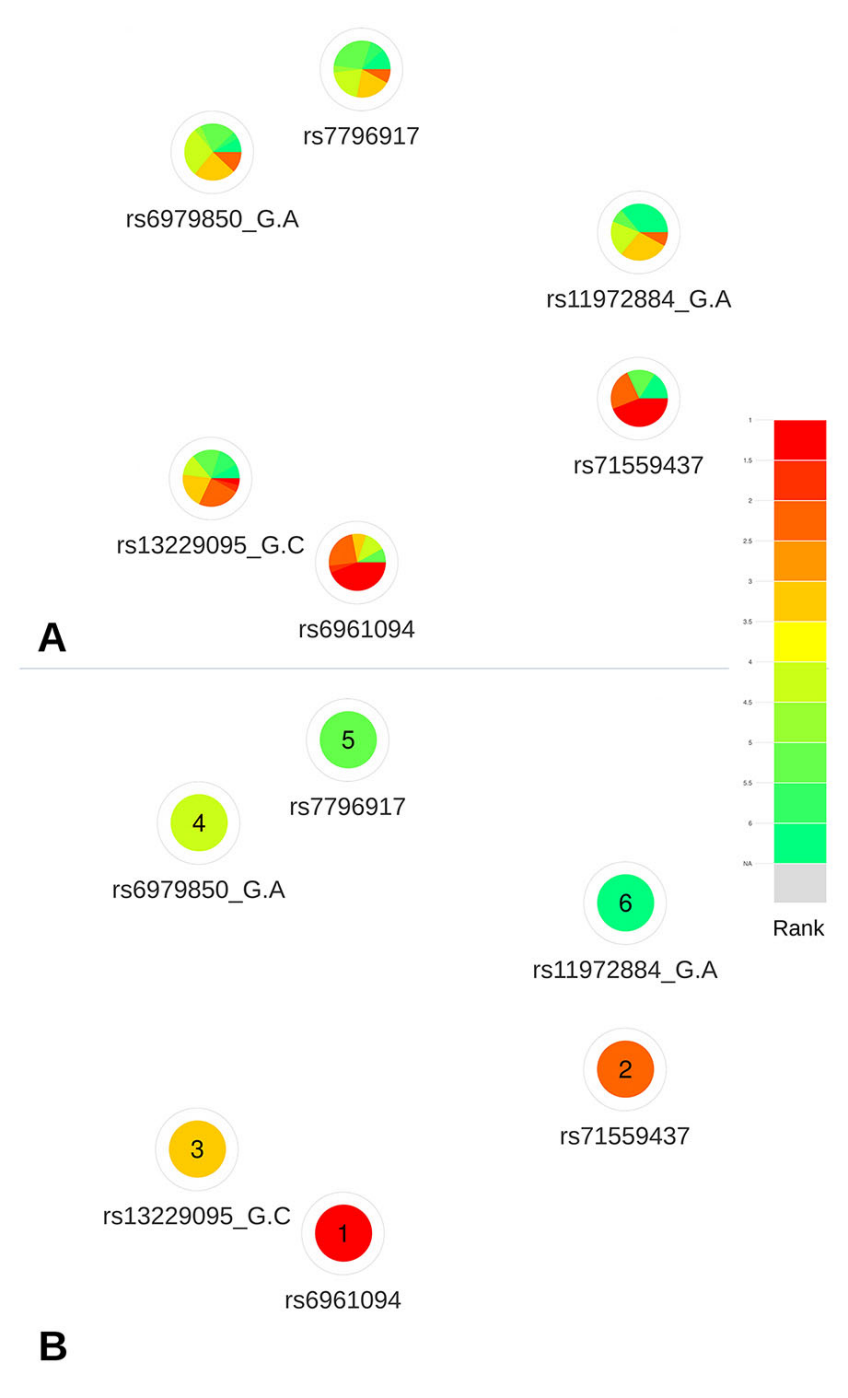
Networks representing the six CRVs associated variants with breast cancer at the 7q22 locus. **(A)** All available predictions represented under the form of relative rank grouped by color. **(B)** Global ranking representing the mean relative ranks with missing values substituted by the median value. Based on the deleteriousness scores available for this subset of variants, a quick overview of variant nodes has allowed to easily identify two variants, rs6961094 and rs71559437, as the best candidates.

Based on the deleteriousness scores available for this subset of variants, a quick overview of variant nodes has allowed to easily identify two variants, rs6961094 and rs71559437, as the best candidates. Indeed, the nodes for these variants contained the largest proportion of red, indicating a good ranking for most of the scoring approaches (**Figure 5A**). The visualization of the mean ranking confirms rs6961094 and rs71559437 as the most credible candidates among the CRVs (**Figure 5B**). These observations are supported by the functional experiments performed by Michailidou et al. (2017), which demonstrated, using allele-specific Chromatin Conformation Capture (3C) assays, that the presence of the risk haplotype (rs6961094 combined with rs71559437) is associated with chromatin looping between *CUX1*, *RASA4*, and *PRKRIP1* promoters suggesting that the protective alleles abrogate this phenomenon.

#### Locus 11p15

This region contains 19 CRVs among 85 candidate variants. Among the 19 CRVs, five variants, located in the proximal promoter of *PIDD1* (a gene implicated in DNA-damage-induced apoptosis and tumorigenesis; Lin et al., 2000), namely, rs7484123, rs7484068, rs11246313, rs11246314, and rs11246316, were further analyzed by Michailidou et al. (2017). They demonstrated, using reporter assays, that these variants, incorporated in a construct, significantly increased *PIDD1* promoter activity.

**Figure 6:**
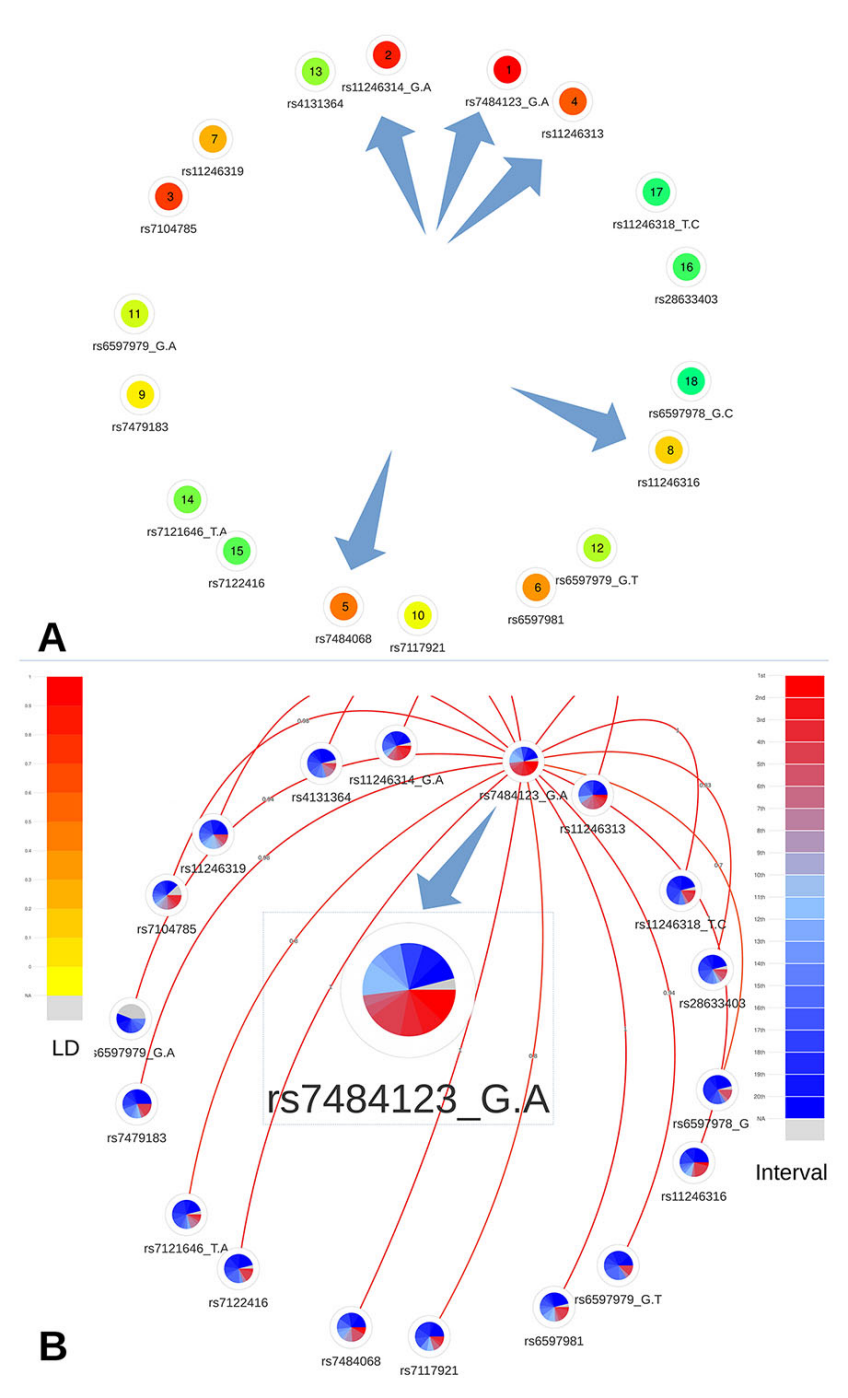
Networks representing the 19 CRVs associated variants with breast cancer at the 11p15 locus. **(A)** Global ranking representing the mean relative ranks with missing values substituted by the median value. The purple arrows highlight the five credible causal variants identified by Michailidou et al. **(B)** The absolute intervals show rs7484123 and rs11246314 as the best candidates with regard to deleteriousness predictions. The best candidate variant rs7484123 sports a high level of linkage disequilibrium (depicted by the red links emanating from rs7484123’s node) with the other candidate variants in the European population.

A quick overview of the relative and absolute metascores visualization allowed to easily prioritize the 19 CRVs (**Figures 6A** and **B**). First, the prioritized list based on the metascores confirms the selection of these five variants as functional credible SNPs. Indeed they are ranked at the first, second, third, fifth, and eighth place out of 19. Moreover, we notice that variants rs7484123 and rs11246314 demonstrate a higher level of coloration, confirming them as the best candidates among the variants located in the proximal promoter of *PIDD1*. The variant rs7484123 particularly stands out as a very promising candidate for subsequent experiments.

### Prioritization of Nine Schizophrenia Susceptibility Loci

As a second example, we have applied DSNetwork to data from an extensive study by Huo et al. (2019) investigating over 180 loci reported to be associated with schizophrenia in several GWAS. This study has prioritized regulatory SNPs at these risk loci using five annotation methods (CADD, Eigen, LINSIGHT, GWAVA, and RegulomeDB) and expression quantitative loci (eQTL) annotation. Potentially causal SNPs have further been identified using functional genomics data such as CHIP-Seq experiments performed on brain tissues. Doing so and using reporter gene assays, they have validated the regulatory effect of nine transcription factor binding-disrupting SNPs from nine different loci.

The list of credible causal variants (CCV) for these nine loci was downloaded from the Psychiatric Genomics Consortium portal (https://www.med.unc.edu/pgc/results-and-downloads/scz/). These regions contained, respectively, 37 CCV on chromosome 1, 73 CCV on chromosome 3, 51 CCV on chromosome 6, 55 CCV on chromosome 7, 32 CCV on chromosome 12, 14 and 5 CCV on chromosome 15, 75 CCV on chromosome 16, and 128 CCV on chromosome 17.

The list of CCV for each locus was uploaded on the DSNetwork tool to identify the best functional candidates. **Table 2** presents, for each of the nine loci, the SNP that was prioritized in the original paper and in the DSNetwork analysis. In cases where results diverged, we also present the ranking provided by DSNetwork for the SNP prioritized in the original paper. From these analyses, we can conclude that DSNetwork found the same top SNP in the majority of cases (five SNPs ranked first and two SNPS ranked in the top 3). Two SNPs ranked in the top 10 but one of them rs696520 was not functionally validated in the original paper. Finally, rs17821573 on the chromosome 16 locus ranked 22nd with DSNetwork. It is important to note that fine-mapping analyses aim at reducing the list of candidate variants and not identifying the causal variant (Cannon and Mohlke, 2018). Furthermore, there is a difference between causal and functional variants: a variant showing a regulatory effect in functional assays does not confirm its implication in a phenotypic variation. Therefore, it would be interesting to test if the top SNP identified by DSNetwork (rs17854029) could also be functional.

**Table 2:** Summarized results from DSNetwork analysis for the nine schizophrenia susceptibility loci.

Locus	# of CCV	Huo et al. top SNP	Validated	DSNetwork top SNP	Huo et al. top SNP in DSNetwork
chr1	37	rs301791	Yes	rs301791	1
chr3	73	rs696520	No	rs9845457	7
chr6	51	rs7752421	Yes	rs7752421	1
chr7	55	rs37718	Yes	rs37718	1
chr12	32	rs7304782	Yes	rs7304782	1
chr15 1	14	rs28676999	No	rs62021888	3
chr15 2	5	rs4702	No	rs4702	1
chr16	75	rs17821573	Yes	rs17854029	22
chr17	128	rs11655813	Yes	rs216172	3
chr17	128	rs9908888	Yes	rs2281727	7

These examples demonstrate the ability of DSNetwork to effectively reduce the amount of CCV despite a large number of candidate variants.

Furthermore, compared to other existing methods for prioritization, DSNetwork has the advantage of being scalable and flexible. Indeed, as a majority voting based approach where each predictor is a crowdsourcing annotator proposing its prioritized list, DSNetwork enables the addition of an infinite number of annotators. However, in practice, one drawback of usual crowdsourcing systems is that the annotators are anonymous. Therefore, their expertise levels are often unknown and uneven, which makes it difficult for the end-user to trust the final vote. In DSNetwork, the annotations are derived from several databases and their reliability level can be estimated through their performance reported in the literature. By default, all the available predictors are used to produce an optimal decision. However, we enable users to adjust the list of predictors used according to their preferences and expertise. As explained in Ribeiro et al. (2016), “explaining the rationale behind individual predictions would make us better positioned to trust or mistrust the prediction, or the classifier as a whole.” For this reason, in order to assist the users in their decision, we provide a short description of each predictor and the list of the annotations they use. Another way to take into account annotator reputation is to add a weight to each vote, the weights representing the competence levels (Tao et al., 2019). This explicit way to incorporate weight in the voting process could be included in further development.

## Conclusion

We analyzed four breast cancer risk loci through DSNetwork and were able to pinpoint the same most plausible causal variants than those proposed in the original paper. In a similar way, we were able to efficiently circumscribe the number of credible candidate variants throughout the prioritization of nine schizophrenia susceptibility loci. DSNetwork provides a user-friendly interface integrating predictors for both coding and non-coding variants in an easy-to-interpret visualization to assist the prioritization process. The use of DSNetwork greatly facilitates the selection process of potentially deleterious variants by aggregating the results of nearly 60 prediction approaches and easily highlighting the best candidate variants for further functional analysis.

## Data Availability Statement

Publicly available datasets were analyzed in this study. This data can be found here: PMID:29059683, PMID:30737407, and https://www.med.unc.edu/pgc/results-and-downloads/scz/.

## Author Contributions

AL designed and implemented DSNetwork software, conducted literature searches, researched data, and selected relevant articles. AL also created figures and tables, and wrote, formatted, and finalized the article for submission. AL, PS, M-PS-B, and RO-C were in charge to test the software and report all bugs. AL, PS, M-PS-B, and RO-C helped to optimize DSNetwork. AL, PS, JS, and AD supervised and reviewed the design of the study. All authors contributed to writing and reviewing the manuscript.

## Funding

The PERSPECTIVE and PERSPECTIVE I&I projects were supported by the Government of Canada through Genome Canada and the Canadian Institutes of Health Research (grant GPH1293344, grant GP1-155865), the Ministere de l’Économie, Science et Innovation du Québec through Genome Quebec and the Quebec Breast Cancer Foundation.

## Conflict of Interest

The authors declare that the research was conducted in the absence of any commercial or financial relationships that could be construed as a potential conflict of interest.
