# Supplementary material for: DSNetwork: An Integrative Approach to Visualize Predictions of Variants’ Deleteriousness
Source: Front Genet. 2020 Jan 17;10:1349. doi: 10.3389/fgene.2019.01349 (PMC6979780; doi:10.3389/fgene.2019.01349)
Supplement: Supplementary file 1 [file DataSheet_1.pdf]

Predictors_description

Score	Id	Range	Ref	Description	Annotations
BayesDel	bayesdel	[-1.3_0.76]	Feng 2017	BayesDel is a combined deleteriousness score defined as a weighted product of likelihood ratios of multiple deleteriousness predictors.	PolyPhen2, SIFT, FATHMM, LRT, Mutation Taster, Mutation Assessor, PhyloP, GERP++, SiPhy
CADD	cadd.consscore;cadd.phred;cadd.rawscore	[0_7];[1_99];[-6_33]	Kircher et al. 2014; Rentzsch et al. 2018	Combined Annotation Dependent Depletion (CADD) is a framework that integrates multiple annotations into one metric by contrasting variants that survived natural selection with simulated mutations.	Gene annotations Gene annotations, Grantham, phastCons, phyloP, GERP++, mirSVR, targetScan, chromHMM, Encode expression, nucleosome position, histone modification, open chromatin, Segway, tOverlapMotifs, TFBS, mutationDensity, nearestMutation, dbscSNV
CDTS	cdts_percentile	[0_100]	di Iulio et al. 2018	CDTS represents sequence constraint across thousands of individuals and is an asset to help interpret noncoding elements in the human genome, prioritize variants and reconsider gene units at a larger scale.	An intraspecies map of sequence constraint for the human species build on 11,257 whole-genome sequences.
DANN	dbnsfp.dann.rankscore	[0_1]	Quang et al. 2015	DANN uses the same feature set and training data as CADD to train a deep neural network (DNN). DNNs can capture non-linear relationships among features and are better suited than SVMs for problems with a large number of samples and features.	Same as CADD
DeepSEA	deepseq_sig_log2	[1_100]	Zhou et al. 2015	DeepSEA predicts genomic variant effects on a wide range of chromatin features at the variant position (Transcription factors binding, DNase I hypersensitive sites, and histone marks in multiple hunman cell types.	Transcription factors binding, DNase I hypersensitive sites, and histone marks in multiple human cell types.
Eigen	eigen_nc;dbnsfp.eigen.pc.raw_rankscore;dbnsfp.eigen.raw_rankscore;eigen_pc_nc	[-2_5];[0_1];[0_1];[-2_5]	Ionita-Laza et al. 2016 Ionita-Laza et al. 2017	Eigen is a spectral approach to the functional annotation of genetic variants in coding and noncoding regions. Eigen makes use of a variety of functional annotations in both coding and noncoding regions (such as made available by the ENCODE and Roadmap Epigenomics projects), and combines them into one single measure of functional importance.	PolyPhen, GERP, ENCODE, genomic element annotation (Ensembl and Roadmap Epigenomics), Furthermore, tissue-specific eQTLs and splicing QTLs (GTEx)
FATHMM	fathmm_nc;dbnsfp.fathmm-	[0_1];	Shihab et al.	FATHMM is capable of predicting the functional	Sequence conservation, nucleotide

Score	Id	Range	Ref	Description	Annotations
	mkl.coding_rankscore;dbnsfp. fathmm.rankscore;dbnsfp. fathmm.xf.coding_rankscore	[0_1]; [0_1]; [0_1]	2015 Rogers et al. 2017	effects of protein missense mutations by combining sequence conservation within hidden Markov models (HMMs), representing the alignment of homologous sequences and conserved protein domains, with "pathogenicity weights", representing the overall tolerance of the protein/domain to mutations. FATHMM-XF (FATHMM with an eXtended Feature set) is an extension of FATHMM which yields highly accurate predictions for SNVs across the entire human genome. FATHMM-XF assigns a confidence score (a p-score) to every prediction, to simplify interpretation, and focus analysis on a subset of high-confidence predictions (cautious classification).	sequence characteristics, genomic features (codons, splice sites, etc.), amino acid features and expression levels in different tissues.
fitCons	cadd.fitcons;fitcons_nc; dbnsfp.gm12878. fitcons_rankscore;dbnsfp.h1. hesc.fitcons_rankscore; dbnsfp.huvec. fitcons_rankscore;dbnsfp. integrated.fitcons_rankscore	[0_1]; [0_0.5]; [0_1]; [0_1]; [0_1]; [0_1]	Gulko et al. 2015	fitCons, the fitness consequences of functional annotation, integrates functional assays (such as ChIP-Seq) with selective pressure inferred using the INSIGHT method. The result is a score ρ in the range [0.0-1.0] that indicates the fraction of genomic positions evincing a particular pattern (or "fingerprint") of functional assay results, that are under selective pressure.	DNase I digestion and sequencing (DNase-seq) data, RNA sequencing (RNA-seq) data and chromatin immunoprecipitation and sequencing (ChIP-seq)
FunSeq2	funseq	[0_1]	Fu et al. 2014	This tool is specialized to prioritize somatic variants from cancer whole genome sequencing. It contains two components : 1) building data context from various resources; 2) variants prioritization.	Inter- and intra-species conservation; loss- and gain-of-function events for transcription-factor binding; enhancer-gene linkages and network centrality
GenoCanyon	dbnsfp.genocanyon. rankscore	[0_1]	Lu, Q. et al. 2015	GenoCanyon is a whole-genome functional annotation approach based on unsupervised statistical learning. It integrates genomic conservation measures and biochemical annotation data to predict the functional potential at each nucleotide.	GenoCanyon, a whole-genome annotation method that performs unsupervised statistical learning using 22 computational and experimental annotations (GERP, PhyloP, open chromatine, histone modification, TFBS)
GERP	cadd.gerp.rs_deleted;cadd. gerp.r;cadd.gerp.n;dbnsfp. gerp...rs_rankscore	[-12.3_6. 17];[-12. 3_6.17];[- 12.3_6. 17];[0_1]	Davydov et al. 2010	GERP identifies constrained elements in multiple alignments by quantifying substitution deficits.	PhastCons; Conservation

Score	Id	Range	Ref	Description	Annotations
Grantham	cadd.grantham;evs.grantham_score	[5_215];[5_215]	Grantham 1974	The Grantham score attempts to predict the distance between two amino acids, in an evolutionary sense. A lower Grantham score reflects less evolutionary distance.	Distance between two amino acids
GWAVA	gwava_region;gwava_tss;gwava_unmatched	[0_1];[0_1];[0_1]	Ritchie et al. 2014 Ritchie et al. 2015 Ritchie et al. 2016	GWAVA is a tool which aims to predict the functional impact of non-coding genetic variants based on a wide range of annotations of non-coding elements (largely from ENCODE/GENCODE), along with genome-wide properties such as evolutionary conservation and GC-content.	Annotations of non-coding elements (largely from ENCODE/GENCODE), along with genome-wide properties such as evolutionary conservation and GC-content.
IW-Scoring	iwscoring_known;iwscoring_novel	[-5_6];[-5_6]	Wang et al. 2018	IW-Scoring is an Integrative Weighted Scoring model to annotate and prioritise functionally relevant noncoding variations. 11 scoring methods were evaluated. An unsupervised spectral approach for subsequent selective integration was applied to produce two linear weighted functional scoring schemas for known and novel variations.	CADD v1.3, DeepSEA, Eigen (Eigen and Eigen-PC), fitCons, FunSeq2, FATHMM-MKL, GWAVA (region, TSS and unmatched scores) and ReMM
LINSIGHT	linsight	[0_1]	Huang et al. 2017	LINSIGHT combines a generalized linear model for functional genomic data with a probabilistic model of molecular evolution to improves the prediction of noncoding nucleotide sites at which mutations are likely to have deleterious fitness consequences, and which therefore are likely to be phenotypically important.	RNA expression level (RNA-seq read depth), chromatin accessibility (DNase-I hypersensitive sites), histone modifications, bound transcription factors (ChIP-seq peaks), gene annotations (e.g., distance to nearest transcription start site, match to known TFBS motif) and comparative genomics (e.g., phyloP25 or phastCons4 scores)
LRT	dbnsfp.lrt.converted_rankscore	[0_1]	Chun and Fay 2009	LRT is a likelihood ratio test capable of accurately identify a subset of deleterious mutations that disrupt highly conserved amino acids within protein-coding sequences, which are likely to be unconditionally deleterious.	Comparative genomics data set of 32 vertebrate species.
M-CAP	dbnsfp.m_cap_score.rankscore	[0_1]	Jagadeesh et al. 2016	Mendelian Clinically Applicable Pathogenicity (M-CAP) Score is the first pathogenicity classifier for rare missense variants in the human genome that is tuned to the high sensitivity required in the clinic.	SIFT, PolyPhen-2, CADD, MutationTaster, MutationAssessor, FATHMM, LRT, MetaLR, and MetaSVM, measures of base-pair, amino acid, genomic region, and gene conservation: RVIS, PhyloP,

Score	Id	Range	Ref	Description	Annotations
					PhastCons PAM250, BLOSUM62, SIPHY, GERP and 298 new features derived from multiple-sequence alignment of 99 primate, mammalian, and vertebrate genomes to the human genome.
MetaLR	dbnsfp.metalr.rankscore	[0_1]	Dong et al. 2015	MetaSVM and MetaLR are ensemble scores are based on 10 component scores (SIFT, PolyPhen-2 HDIV, PolyPhen-2 HVAR, GERP++, MutationTaster, Mutation Assessor, FATHMM, LRT, SiPhy, PhyloP) and the maximum frequency observed in the 1000 genomes populations.	PolyPhen-2, SIFT, MutationTaster, Mutation Assessor, FATHMM, LRT, SiPhy, GERP++ and PhyloP
MetaSVM	dbnsfp.metasvm.rankscore	[0_1]	Dong et al. 2015	MetaSVM and MetaLR are ensemble scores are based on 10 component scores (SIFT, PolyPhen-2 HDIV, PolyPhen-2 HVAR, GERP++, MutationTaster, Mutation Assessor, FATHMM, LRT, SiPhy, PhyloP) and the maximum frequency observed in the 1000 genomes populations.	PolyPhen-2, SIFT, MutationTaster, Mutation Assessor, FATHMM, LRT, SiPhy, GERP++ and PhyloP
MPC	dbnsfp.mpc.rankscore	[0_1]	Samocha et al. 2017 (bioRxiv)	MPC (for Missense badness, PolyPhen-2, and Constraint) is a novel missense deleteriousness metric which specifically enables the deleterious effect of missense variants to be predicted.	Map of sub-genic regions depleted of missense variations based on the exome sequencing data of 60,706 individuals from (ExAC)
MutationAssessor	dbnsfp.mutationassessor. rankscore	[0_1]	Reva et al. 2011	MutationAssessor predicts the functional impact of amino-acid substitutions in proteins, such as mutations discovered in cancer or missense polymorphisms. The functional impact is assessed based on evolutionary conservation of the affected amino acid in protein homologs.	Sequence homology of protein families and sub-families within and between species
MutationTaster2	dbnsfp.mutationtaster. converted_rankscore	[0_1]	Schwarz et al. 2014	MutationTaster2 evaluates the pathogenic potential of DNA sequence alterations. It is designed to predict the functional consequences of not only amino acid substitutions but also intronic and synonymous alterations, short insertion and/or deletion (indel) mutations and variants spanning intron-exon borders.	DNA sequence conservation, splice site prediction, mRNA stability prediction and protein feature annotations
MutPred	dbnsfp.mutpred.rankscore; mutdb.mutpred_score	[0_1]; [0_1]	Li et al. 2009	MutPred2 is a machine learning-based method and software package that integrates genetic and molecular data to reason probabilistically about the pathogenicity of amino acid substitutions. This is achieved by providing (1) a general	MutPred2 is a machine learning-based method and software package that integrates genetic and molecular data to reason probabilistically about the pathogenicity of amino acid

Score	Id	Range	Ref	Description	Annotations
				pathogenicity prediction, and (2) a ranked list of specific molecular alterations potentially affecting the phenotype.	substitutions.
PhastCons	cadd.phast_cons.mammalian; cadd.phast_cons.primate; cadd.phast_cons.vertebrate; dbnsfp.phastcons.100way. vertebrate_rankscore;dbnsfp. phastcons.20way. mammalian_rankscore	[0_1]; [0_1]; [0_1]; [0_1]; [0_1]	Siepel et al. 2005	PhastCons is a program for identifying evolutionarily conserved elements in a multiple alignment, given a phylogenetic tree. It is based on a statistical model of sequence evolution called a phylogenetic hidden Markov model (phylo-HMM).	Distributions of the number of substitutions based on a phylogenetic hidden Markov model
PhyloP	cadd.phylop.mammalian; cadd.phylop.primate;cadd. phylop.vertebrate;dbnsfp. phylo.p100way. vertebrate_rankscore;dbnsfp. phylo.p20way. mammalian_rankscore	[-14_3];[-14_3];[-14_3]; [0_1]; [0_1]	Siepel et al. 2006	PhyloP scores measure evolutionary conservation at individual alignment sites. The scores are interpreted as follows compared to the evolution expected under neutral drift: Positive scores -- Measure conservation, which is slower evolution than expected, at sites that are predicted to be conserved.	Sequences under selection based on comparative sequence based on a phylogenetic hidden Markov model
PolyPhen2	dbnsfp.polyphen2.hdiv. rankscore;dbnsfp.polyphen2. hvar.rankscore	[0_1]; [0_1]	Adzhubei et al. 2010	PolyPhen-2 (Polymorphism Phenotyping v2) is a tool which predicts possible impact of an amino acid substitution on the structure and function of a human protein using straightforward physical and comparative considerations. Please, use the form below to submit your query.	Amino acid substitution on the structure and function of a human protein.
PROVEAN	dbnsfp.provean.rankscore	[0_1]	Choi et al. 2012	PROVEAN (Protein Variation Effect Analyzer) is a software tool which predicts whether an amino acid substitution or indel has an impact on the biological function of a protein.	Alignment-based score predicting the damaging effects of single amino acid substitutions, in-frame insertions, deletions, and multiple amino acid substitutions.
ReMM	remm	[0_1]	Smedley et al. 2016	The Regulatory Mendelian Mutation (ReMM) score was created for relevance prediction of non-coding variations (SNVs and small InDels) in the human genome (hg19) in terms of Mendelian diseases.	PhastCons, PhyloP, GERP++, CpG and G/C content, transcription and regulation annotations from UCSC, DNase hypersensitive score, Histone methylation, TFBS, MAF, DGV, dbVar and isca
REVEL	dbnsfp.revel.rankscore	[0_1]	Ioannidis et al. 2016	REVEL is (rare exome variant ensemble learner) an ensemble method for predicting the pathogenicity of missense variants on the basis of individual tools such as MutPred, FATHMM, VEST and PolyPhen.	MutPred, PROVEAN, SIFT, PolyPhen-2 HVAR and HDIV, LRT, MutationTaster, MutationAssessor, FATHMM v2.3, and VEST 3.0, GERP++, SiPhy, phyloP, phastCons, PolyPhen-2, FATHMM, and

Score	Id	Range	Ref	Description	Annotations
					PROVEAN.
SIFT	cadd.sift.val;dbnsfp.sift.converted_rankscore	[1_0]; [0_1]	Ng and Henikoff 2003	SIFT (sorting intolerant from tolerant) predicts whether an amino acid substitution affects protein function based on sequence homology and the physical properties of amino acids. SIFT can be applied to naturally occurring nonsynonymous polymorphisms and laboratory-induced missense mutations.	Distribution of amino acid residues observed at a given position in the sequence alignment and the estimated unobserved frequencies of amino acid distribution calculated from a Dirichlet mixture.
SIFT4G	dbnsfp.sift4g.converted_rankscore	[0_1]	Vaser R et al. 2016	SIFT 4G is faster version of SIFT that enables practical computations on reference genomes.	Distribution of amino acid residues observed at a given position in the sequence alignment and the estimated unobserved frequencies of amino acid distribution calculated from a Dirichlet mixture.
SiPhy	dbnsfp.siphy_29way.logodds_rankscore	[0_1]	Garber et al. 2009; Lindblad-Toh et al. 2011	SiPhy is a statistical method for modeling biased nucleotide substitutions, a learning algorithm for inferring site-specific substitution biases directly from sequence alignments and a hidden Markov model for detecting constrained elements characterized by biased substitutions.	Deeply sequenced clades to identify evolutionary selection and substitution patterns characteristic of sequence undergoing natural selection.
VEST	dbnsfp.vest3.rankscore; dbnsfp.vest4.rankscore	[0_1]; [0_1]	Carter et al. 2013	VEST (Variant Effect Scoring Tool) is a machine learning method that predicts the functional significance of missense mutations based on the probability that they are pathogenic.	Functional missense mutations
