# Supplementary material for: DSNetwork: An Integrative Approach to Visualize Predictions of Variants’ Deleteriousness
Source: Front Genet. 2020 Jan 17;10:1349. doi: 10.3389/fgene.2019.01349 (PMC6979780; doi:10.3389/fgene.2019.01349)
Supplement: Supplementary file 2 [file DataSheet_2.pdf]

User guide

Visualization concept

The DSNetwork approach was designed to assist researchers in the prioritization process of functional variants derived from GWAS association tests. Indeed, this type of analysis pinpoints genetic loci statistically associated to a particular phenotypic trait. These loci may contain several independent signals and these signals may themselves include hundreds of genetic variants associated with the trait. Those variants being sometimes statistically indistinguishable, it becomes essential to add exogenous knowledge to help identify potential functional causal variants among the background variants. Among the available methods, many are based on the evaluation or the prediction of functional impact of variants. However, the multiplication of approaches as well as their different implementations makes the interpretation of the predictions and the decision making very challenging.

In this context, we created a web application called DSNetwork for **D**ecision **S**upport **N**etwork. This tool aims to provide the users with deleteriousness predictions for human variants (hg19 build) recovered from several sources, and to present these scores in a user-friendly web interface.

The following paragraphs describe DSNetwork's approach through the hypothetical analysis of a loci containing 5 variants rs4233486, rs35054111, rs11808410, rs11804913 and rs7554973 using the deleteriousness scores of 5 distinct fictive predictors namely A, B, C, D and E. Table 1 summarizes the scores generated by these 5 predictors, reflecting their predictions regarding the functional impacts of the candidate variants.

DSNetwork integrates the characteristics of the different predictors and creates a reference frame containing the lower and upper boundaries as well as the direction (ascending [ASC], or descending [DESC]) of their prediction scores (Figure 1). The direction is used to rank variants from the most deleterious to the least deleterious on the basis of their respective scores. The boundaries are used to establish the absolute deleteriousness level of each variant.

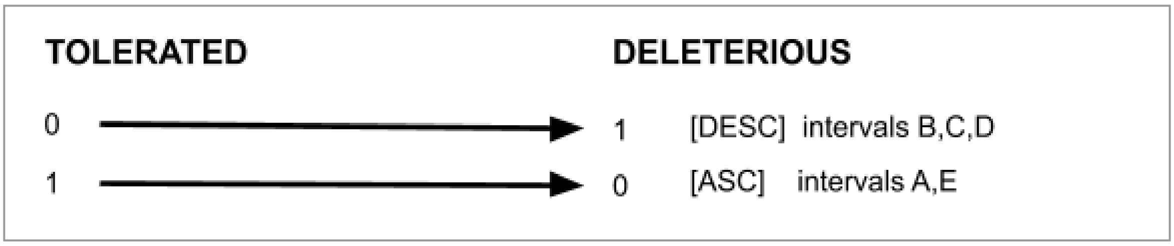

Figure 1: Predictor reference frame

Once the different reference frames are integrated, they can be used to prioritize the variants according to 3 types of representations: the intra-predictor relative ranks, the intra-predictor absolute scores and the global ranks.

Here are the scores obtained for the 5 candidate variants for these 5 approaches:

	A	B	C	D	E
**rs4233486**	0.13	0.40	0.78	0.23	0.12
**rs35054111**	NA	0.70	0.21	NA	0.43
**rs11808410**	0.51	0.40	0.21	0.20	0.77
**rs11804913**	0.01	0.40	0.21	0.30	0.37
**rs7554973**	0.20	0.5	0.55	NA	0.01

Table 1: Annotations for the five candidate variants

Intra-predictor ranks

Intra-predictor ranks allow the prioritization of a list of variants relative to one another. According to the reference frames illustrated in Figure 1, the 5 predictors produce scores ranging from 0 to 1. We can classify the 5 variants of interest from the most deleterious (rank 1) to the least deleterious (rank 5) with each predictor (Table 2).

	A	B	C	D	E
**rs4233486**	2	4	1	2	2
**rs35054111**	NA	1	4	NA	4
**rs11808410**	4	4	4	3	5
**rs11804913**	1	4	4	1	3
**rs7554973**	3	2	2	NA	1

Table 2: Intra-predictor ranking

In order to summarize this information in an easy-to-interpret representation, each variant is depicted as a pie chart where each slice represents the rank of the variant for one of the predictors. Thus, in the current analysis, five pie charts are generated and each pie chart is divided into five slices of the same size. The slices are ordered by predictor by default.

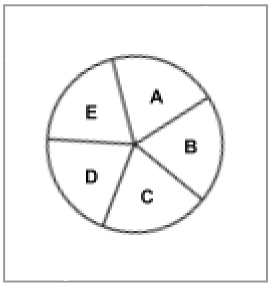

Figure 2: Pie chart and slice distribution

We used a color gradient ranging from red to green, where red corresponds to the most deleterious variant (rank 1) among the candidates for a given predictor. The gray color represents missing data. Figure 3 depicts the pie charts generated for the five candidate variants. The slices can be ordered by color to allow easy identification of variants that appear the most deleterious across predictors.

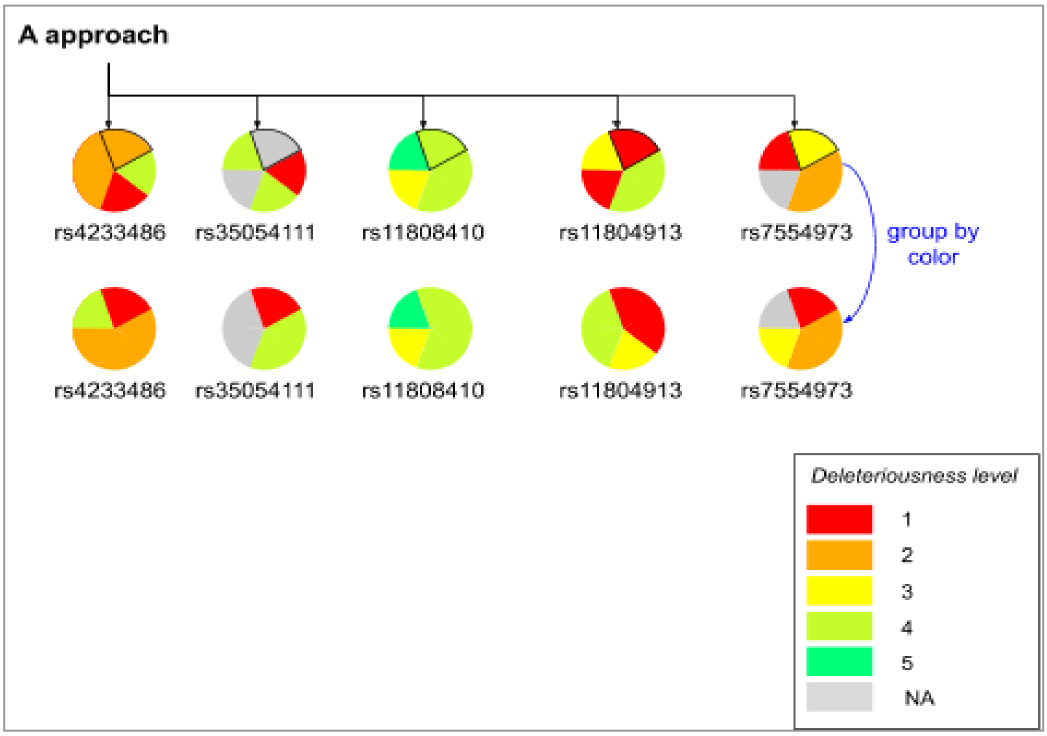

Figure 3: Visualization of intra-predictor ranking

Intra-predictor absolute scores

Intra-predictor absolute scores allow prediction of variant deleteriousness in reference to the thresholds established for a particular predictor. Given these boundaries, we can determine where each variant is located on the deleteriousness spectrum for each predictor. We chose to divide the score range of each approach into 20 equal intervals. The first interval contains the most deleterious scores and the 20th, the least deleterious. Thus, the annotation scores obtained for each variant are translated into their corresponding intervals. This allows the user to know if a variant is predicted as deleterious by a particular approach without having to know the implementation details of this approach. For clarity purposes, in this example the range of scores has been divided into 4 intervals (instead of 20) (Table 3).

	A	B	C	D	E
**rs4233486**	1	3	1	4	1
**rs35054111**	NA	2	4	NA	2
**rs11808410**	3	3	4	4	4
**rs11804913**	1	3	4	3	2
**rs7554973**	1	3	2	NA	1

Table 3: Intra-predictor intervals

As for intra-predictor ranks, each variant is depicted as a pie chart where each slice represents the score interval of the variant for a particular predictor.
We used a color gradient ranging from red to blue. The red color represents the most deleterious interval for a given predictor. The gray color represents missing data. Figure 4 depicts the pie charts

generated for the five candidate variants. The slices can be ordered by color to easily identify variants with the most predictions of deleteriousness.

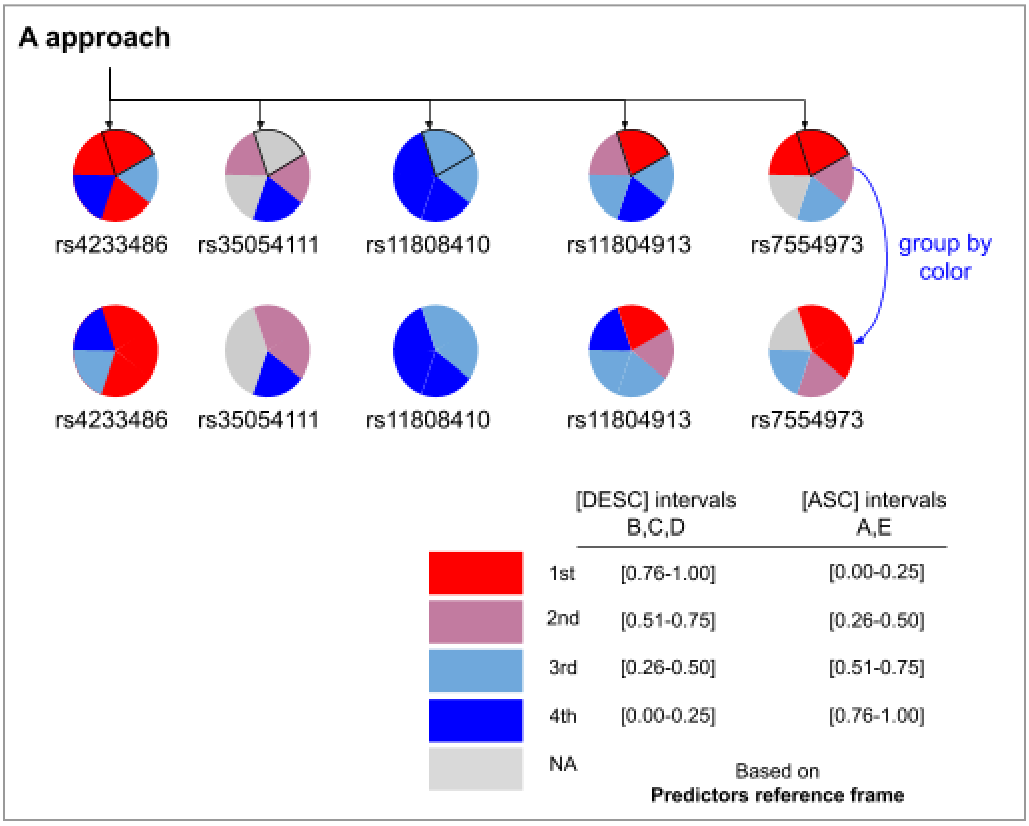

Figure 4: Visualization of intra-predictor scores intervals

Global ranking

In order to further facilitate the prioritization, we propose to summarize the information regarding the relative ranks in an overall rank for each variant.

To do so, we calculate the average rank of each variant based on its intra-predictor ranks. Then, we order the variants according to their average rank. Variants with the lowest average ranks are considered as the best candidates for being deleterious. Because in some cases there may be missing values for some of the predictors when analysing a specific set of variants, we propose three strategies for calculating a consistent average rank which will be comparable between variants and which will take into account these missing values: 1) replace missing values with the average value (default one, Table 4); or 2) replace missing values with the median value (Table 5). Once the necessary substitutions are made, the average ranks can be calculated and the global ranks generated.

	**Score A**	**Rank with missing value exclusion**	**Score A with mean substitution**	**Rank with mean substitution**
**rs4233486**	0.13	2	0.13	2
**rs35054111**	NA	NA	0.2125	4
**rs11808410**	0.51	4	0.51	5

**rs11804913**	0.01	1	0.01	1
**rs7554973**	0.20	3	0.20	3

Table 4: Mean-based substitution

	**Score A**	**Rank with missing value exclusion**	**Score A with median substitution**	**Rank with median substitution**
**rs4233486**	0.13	2	0.13	2
**rs35054111**	NA	NA	0.165	3
**rs11808410**	0.51	4	0.51	5
**rs11804913**	0.01	1	0.01	1
**rs7554973**	0.20	3	0.20	4

Table 5: Median-based substitution

Once the necessary substitutions have been made, the average ranks can be calculated and the global ranks generated (Table 6).

	**A**	**B**	**C**	**D**	**E**	**Mean rank**	**rank**
**rs4233486**	2	4	1	3	2	2.4	1.5
**rs35054111**	3	1	4	3	4	3.0	4
**rs11808410**	5	4	4	5	5	4.6	5
**rs11804913**	1	4	4	1	3	2.6	3
**rs7554973**	4	2	2	3	1	2.4	1.5

Table 6: Global ranking

As for the intra-predictor scores and ranks, the global ranks are made available for each variant under the form of a pie chart where the rank is represented by a color gradient ranging from red to green. The color red represents the most deleterious variant among the candidates for all approaches (Figure 5).

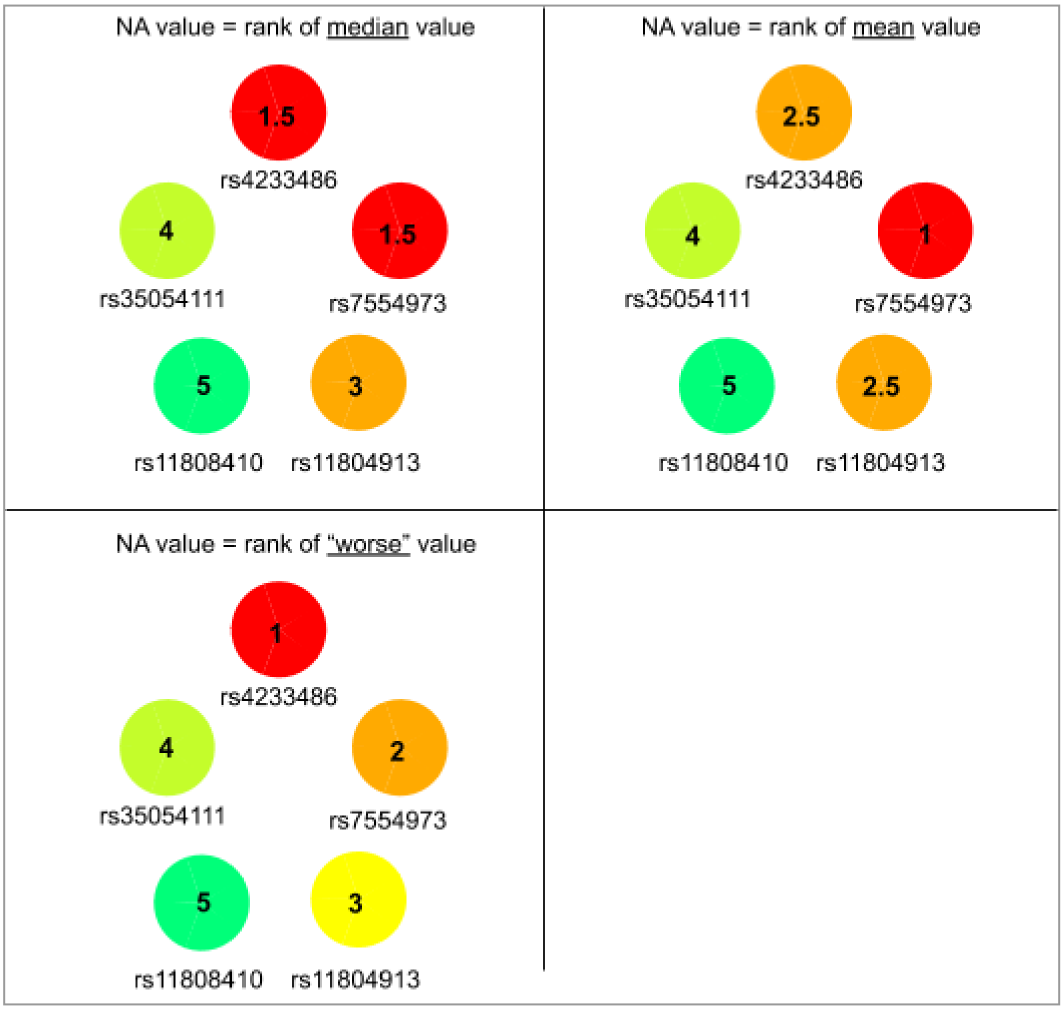

Figure 5: Global ranking representation according to the substitution method

Variants network

DSNetwork offers the possibility to simply visualize scores and linkage disequilibrium between variants in order to identify potential haplotypes. The scores are the nodes of the network and the LD between the different variants is represented by the links between the nodes. The level of LD is estimated via the r2 measure and represented in a color gradient ranging from yellow to red. The red representing a total imbalance is r2 = 1. The gray color represents the missing information (Figure 6).

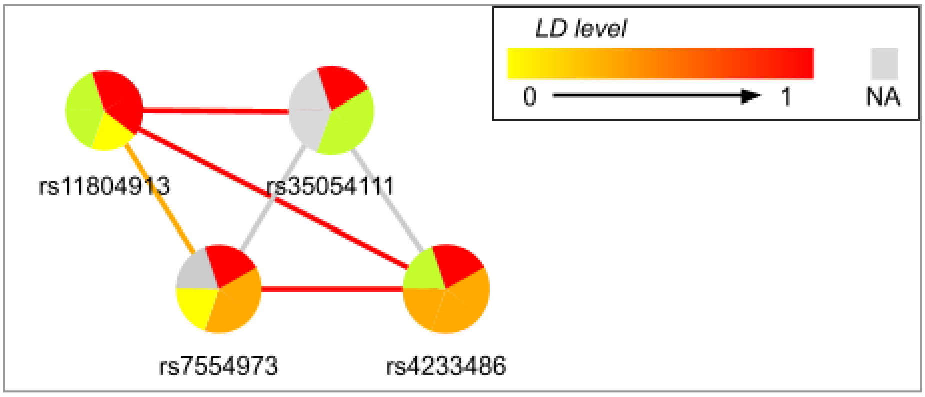

Figure 6: Linkage disequilibrium between variants

Conclusion

Considering the relative ranks, the two best candidate variants of our hypothetical analysis are rs4233486 and rs7554973. Indeed, depending on the substitution approach, these two variants are respectively first, first ex aequo or second among the 5 analysed variants. However, despite an apparent draw, rs4233486 could be the best candidate when taking into account the absolute score intervals. For three of the five approaches, rs4233486 is found in the most deleterious intervals. However, one cannot exclude the putative functional impact of rs7554973 with regard to its scores and the high LD with rs4233486.

Tool usage

The application is divided into 3 panels : 1) Input, 2) Selection, 3) Visualization.

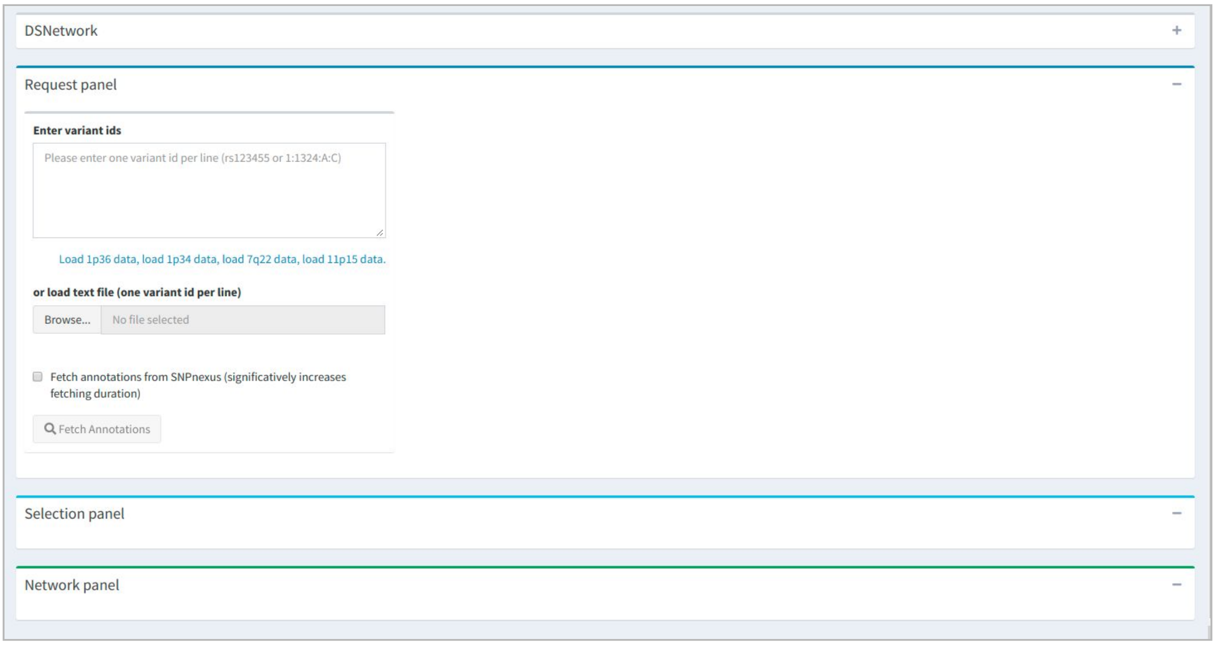

Input

Users can analyse a specific set of variants either by pasting the SNP list in the text area provided for this purpose or by uploading a text file. This file should contain only the SNP list with one identifier per line.
Variants should be referenced through their dbSNP (Sherry et al. 2001) identifiers or their genomic positions and alleles (e.g 5:44527739:A:ATACT). The selected variants must be located on the same chromosome.
Once the variant list is uploaded, the ”Fetch annotations” button will trigger the score retrieval process.

Either paste your variants list in the text area ...

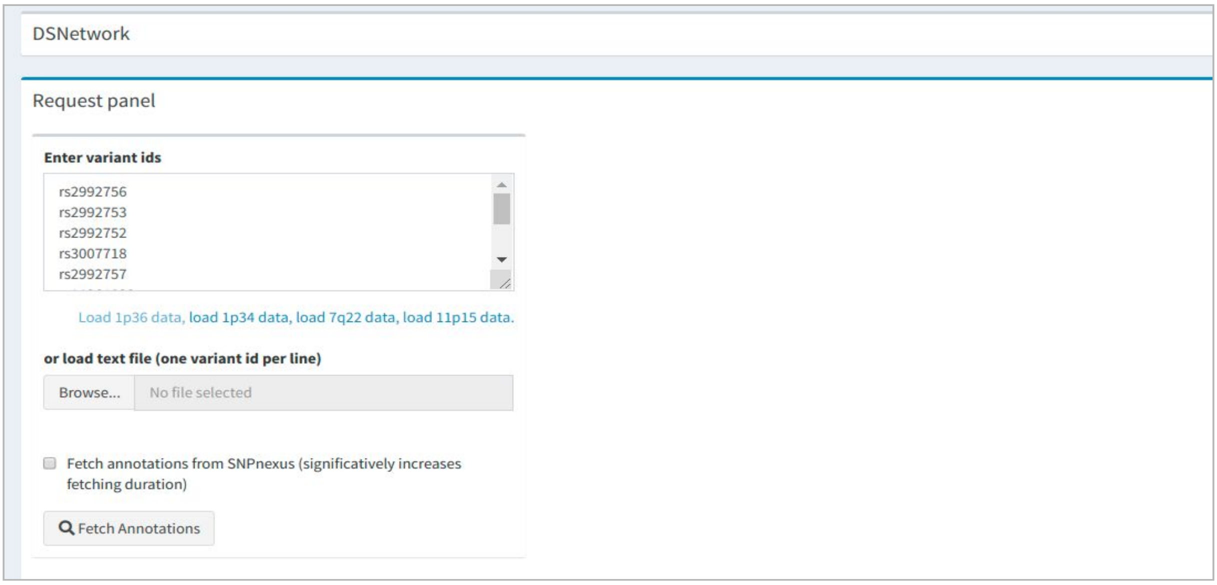

or upload a text file containing a variant id per line.

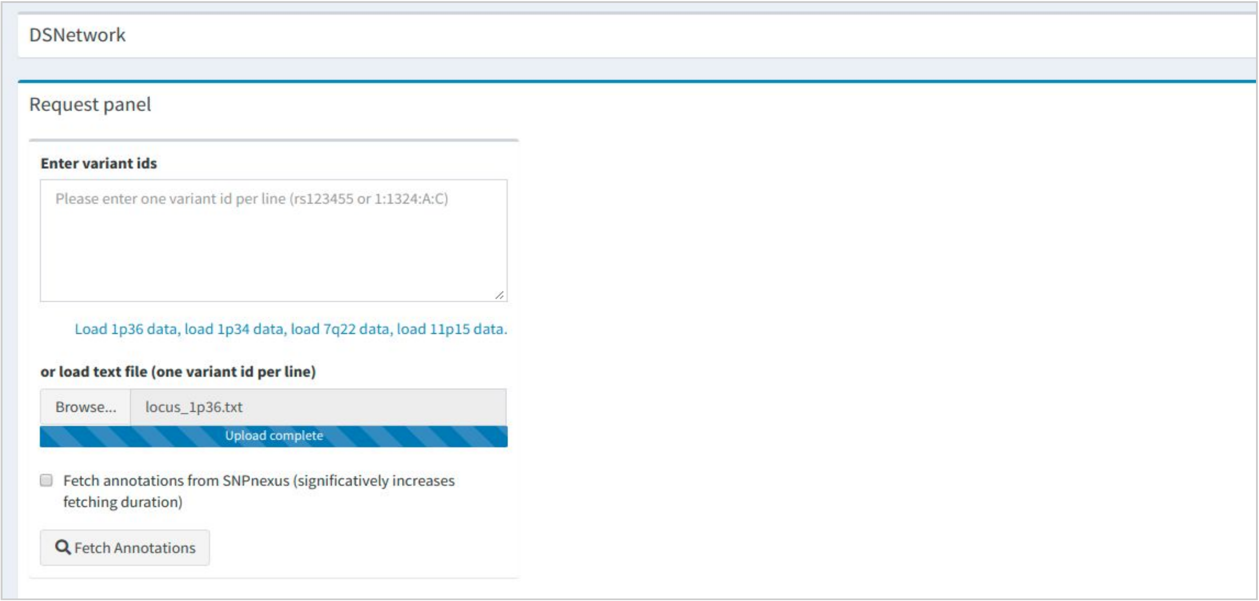

Once your data has loaded, you may choose to trigger SNPNexus data retrieval. As the data needs to be retrieved from the server, a certain processing time should be expected. You can configure the waiting time (in minutes) through a slide bar.

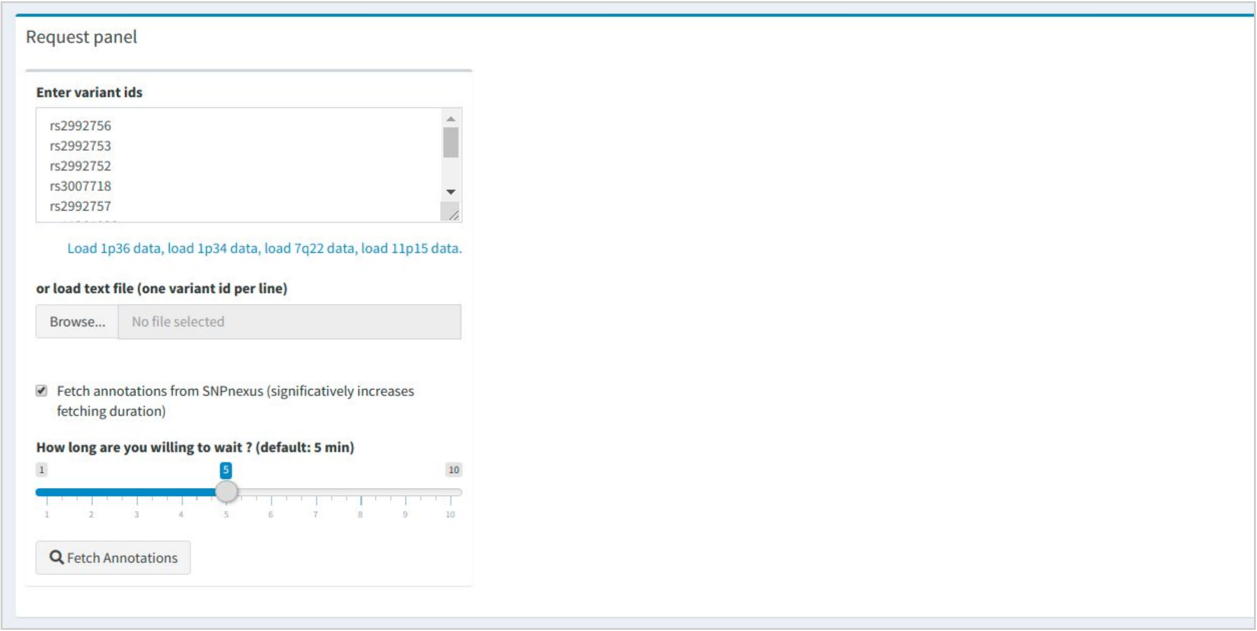

Press "Fetch annotations" to trigger annotation retrieval.

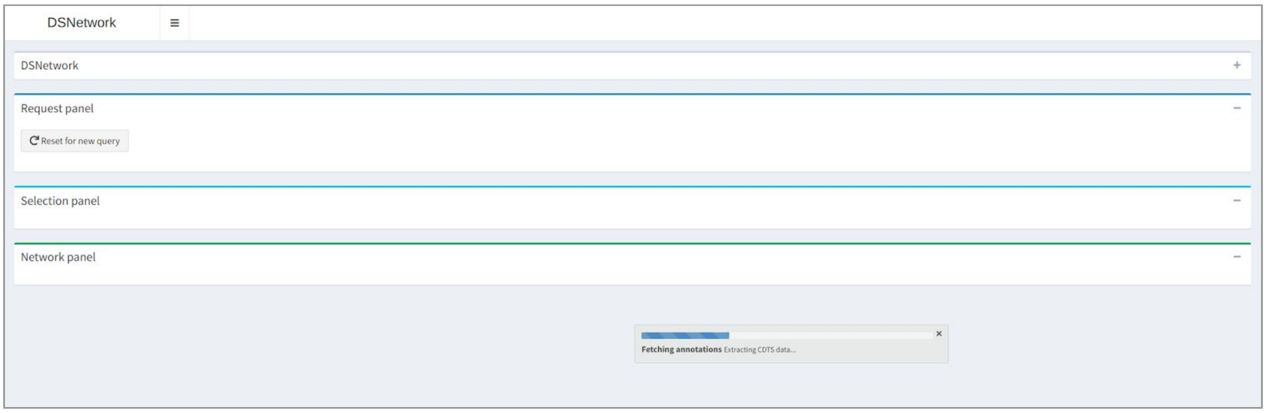

An overview of the results is presented in a scatter plot representing the requested variants along the map of sequence constraint **C**ontext-**D**ependent **T**olerance **S**core (CDTS) - determined through alignment of genomes from thousands of individuals.

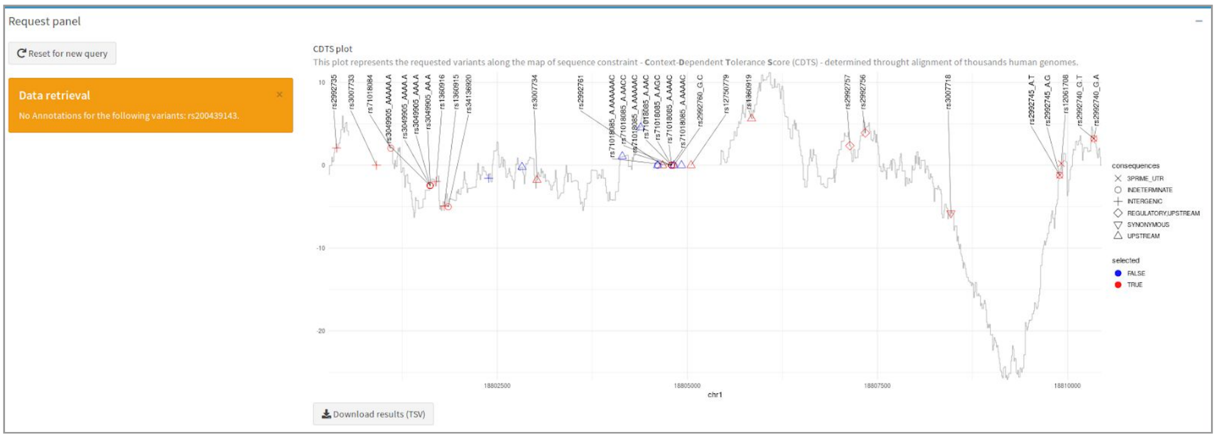

Legend :

- Color code: selected variants, unselected variants
- Shape code enables to distinguish variants according to their regulatory consequences. 7 different shapes are available. If there is more than 7 different annotations, the rarer annotations will all have the same shape, the square.

By default, the best variants with regards to the overall global mean ranking are selected (up to 30).

You can download all the annotations by pressing “Download results (TSV)”

Selection

In the second panel, for consistency purposes, non-synonymous and regulatory variants are processed separately. Once the annotations are fetched, a summary table will appear in the selection panel. Without user specific selection, the first regulatory variants, up to a maximum of thirty, will be highlighted in the table and CDTS plot.

The summary table contains 6 columns: query, HGVS ids, CADD consequences and 3 columns containing overall global mean ranks (OGMR) according to the three substitution approaches.

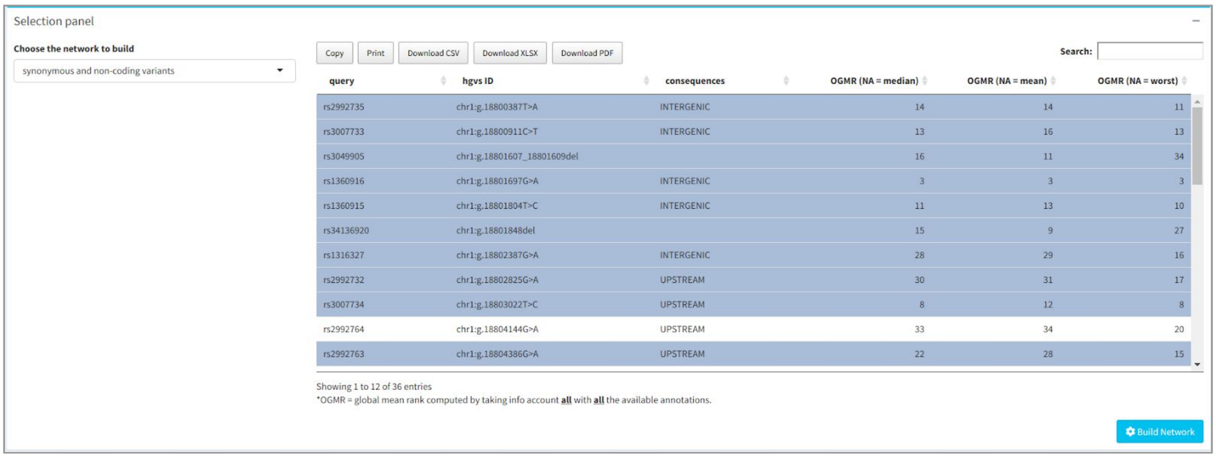

query	hgvs ID	consequences	OGMR (NA = median)	OGMR (NA = mean)	OGMR (NA = worst)
rs2992735	chr1:g.18800387T>A	INTERGENIC	14	14	11
rs3007733	chr1:g.18800911C>T	INTERGENIC	13	16	13
rs3049905	chr1:g.18801607_18801609del		16	11	34
rs1360916	chr1:g.18801697G>A	INTERGENIC	3	3	3
rs1360915	chr1:g.18801804T>C	INTERGENIC	11	13	10
rs34136920	chr1:g.18801848del		15	9	27
rs1316327	chr1:g.18802387G>A	INTERGENIC	28	29	16
rs2992732	chr1:g.18802825G>A	UPSTREAM	30	31	17
rs3007734	chr1:g.18803022T>C	UPSTREAM	8	12	8
rs2992764	chr1:g.18804144G>A	UPSTREAM	33	34	20
rs2992763	chr1:g.18804386G>A	UPSTREAM	22	28	15

Showing 1 to 12 of 36 entries
*OGMR = global mean rank computed by taking info account **all** with **all** the available annotations.

⚠ Please notice that OGMR are computed by taking into account <u>all the variants</u> and <u>all the available annotations</u> and are <u>not updated</u> when some variants or annotations are excluded from the analysis in the following steps.

Users can change the type of variants to visualise through the dropdown list at the right of the selection panel.

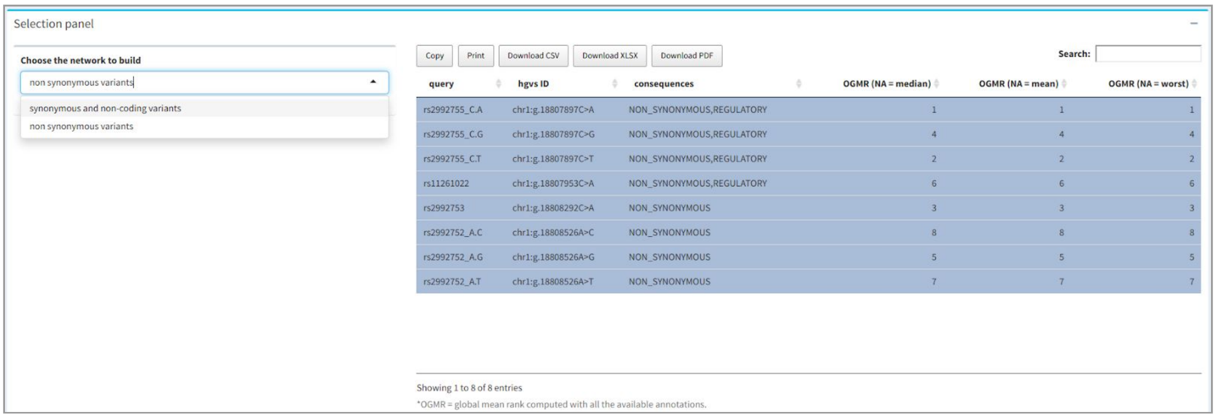

Which will automatically update the CDTS plot and highlight the preselected coding variants.

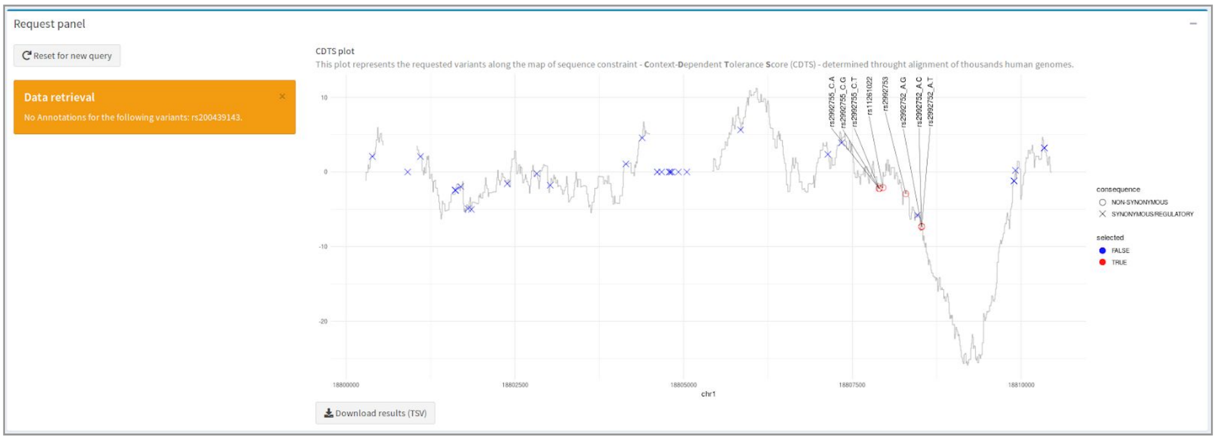

You can also change the selected variants through this table.
The number of selectable variants is restricted to 30 for ergonomic reasons.

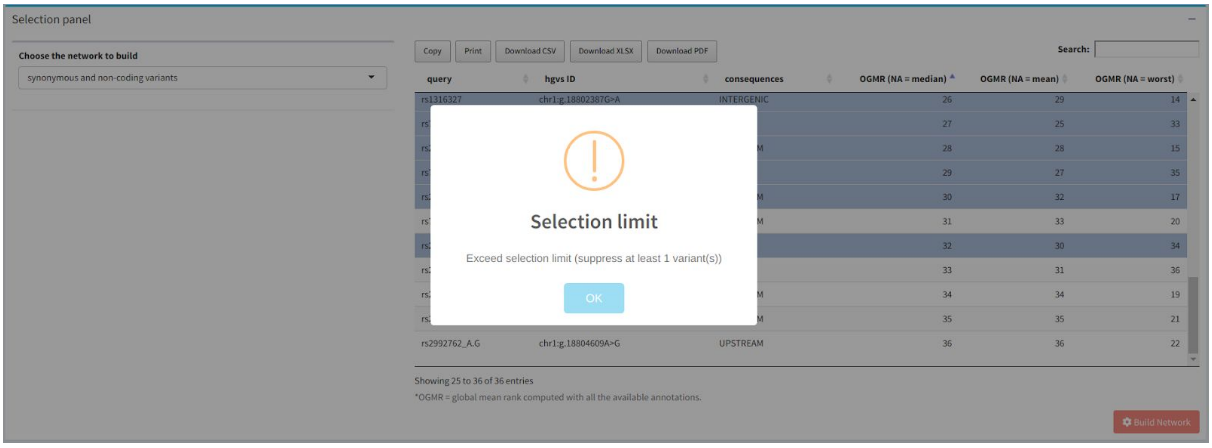

You can use the reported global scores in the summary table to select a particular subset of variants. In the following picture, we selected regulatory variants which global rank is inferior to 5 with one of the substitution approaches.

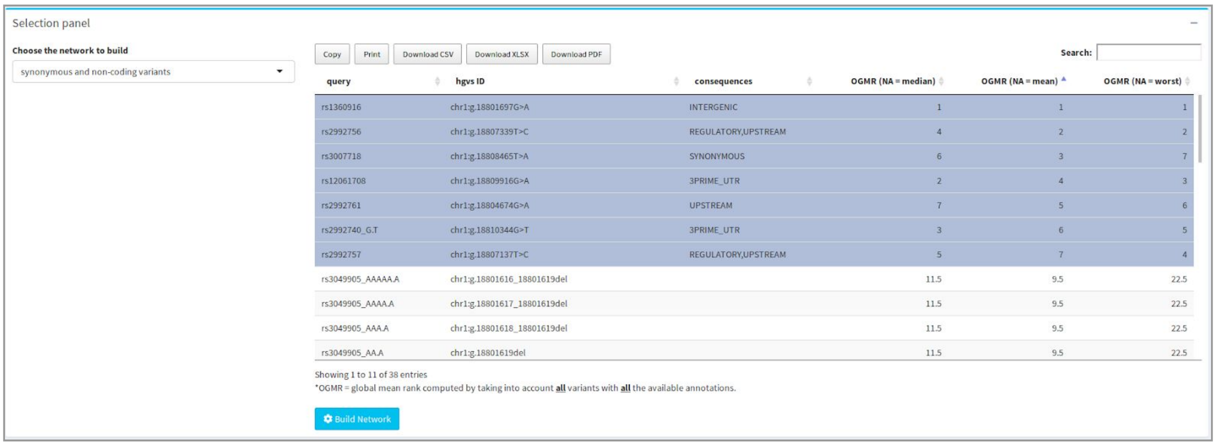

It will automatically load the CDTS plot.

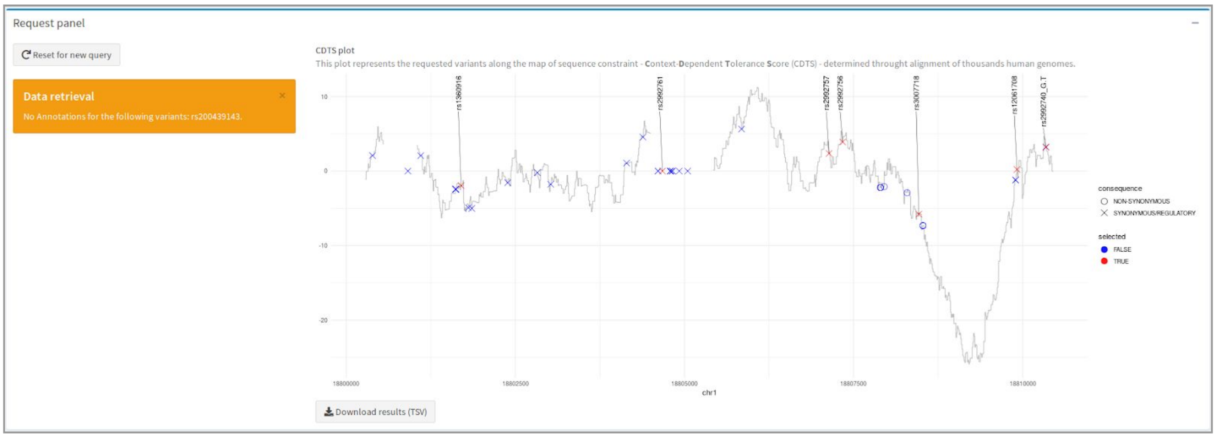

⚠ The automatic update of the CDTS plot can affect the responsiveness of the selection table. To avoid this behaviour, collapse the “Request panel” by clicking on the minus sign located at the top right.

Visualization

Once the variants are selected, press “Build Network” to build the decision support network.
By default, the relative intra-predictor ranks - ordered by predictors - are displayed.

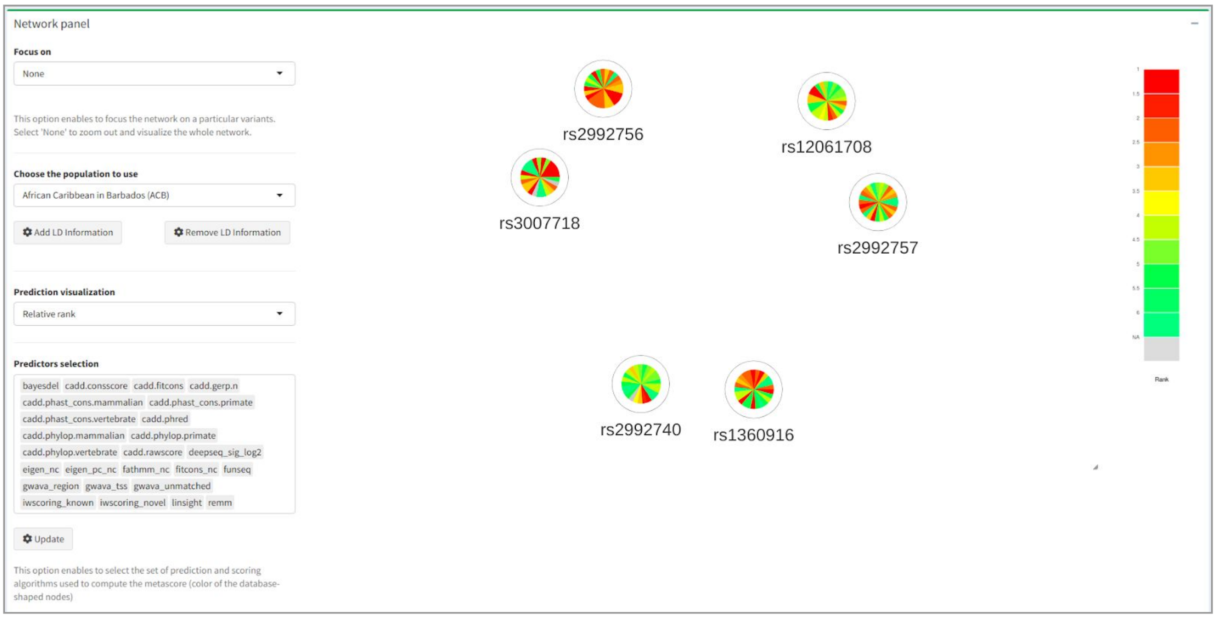

Interactions with the network

Once your mouse is the network area, you can use it to interact with the network in the following matter:

- Scroll in or out to zoom

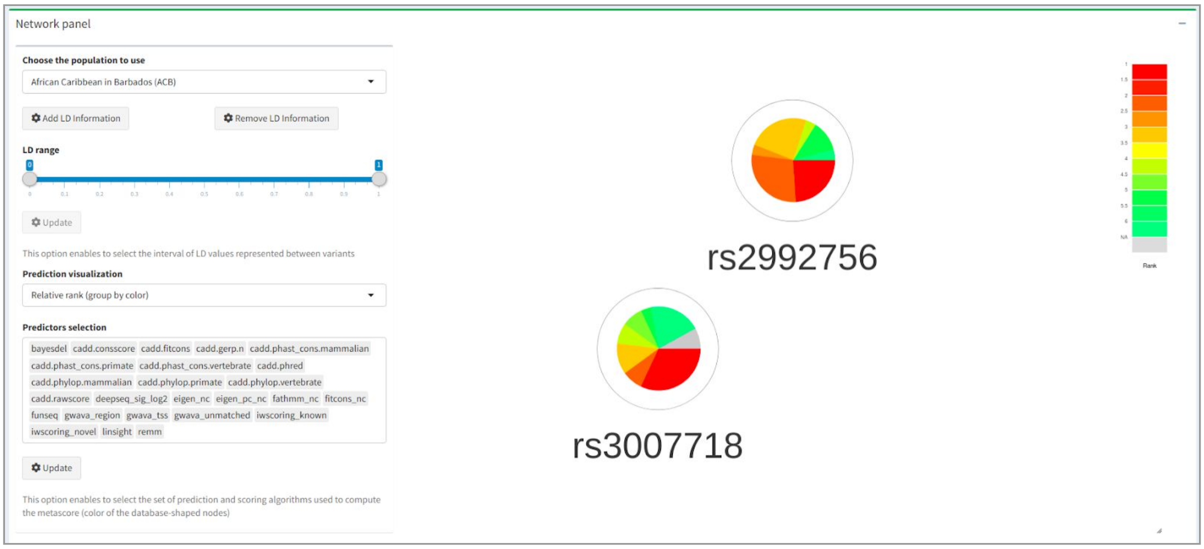

- Drag and drop to move the network within the network area
- Grab the right bottom border and drag to the desired width or height to adjust the size of the network
- Double click on a variant pie chart to get variant annotation details

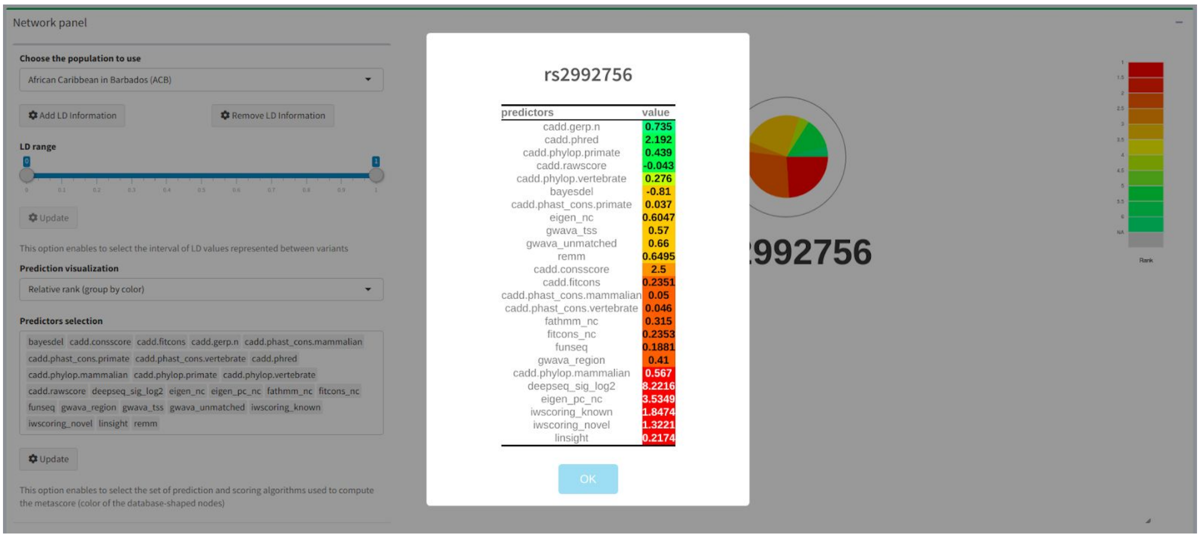

- Right click in the network area and select the “Save image as” option to save the network image in “png” format

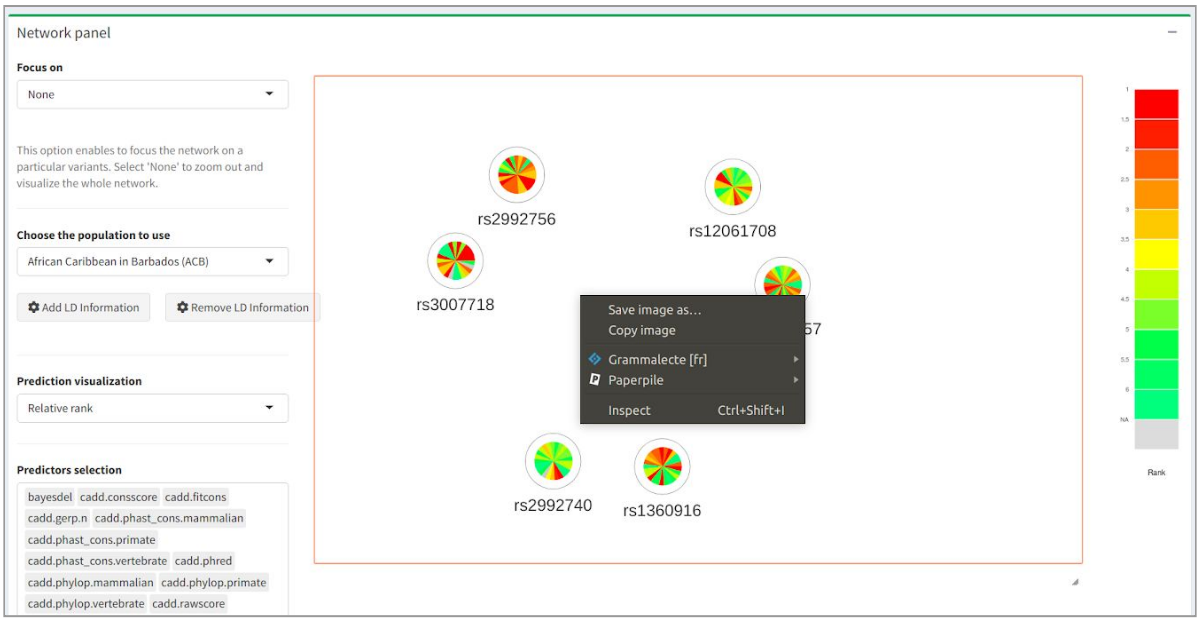

Visualization parameters are available at the left of the Network panel and will be described from the top to the bottom.

Focus

This first parameter enables to automatically focus the network on a particular variant. This is a useful option when dealing with big networks. The "none" option restores the initial visualization.

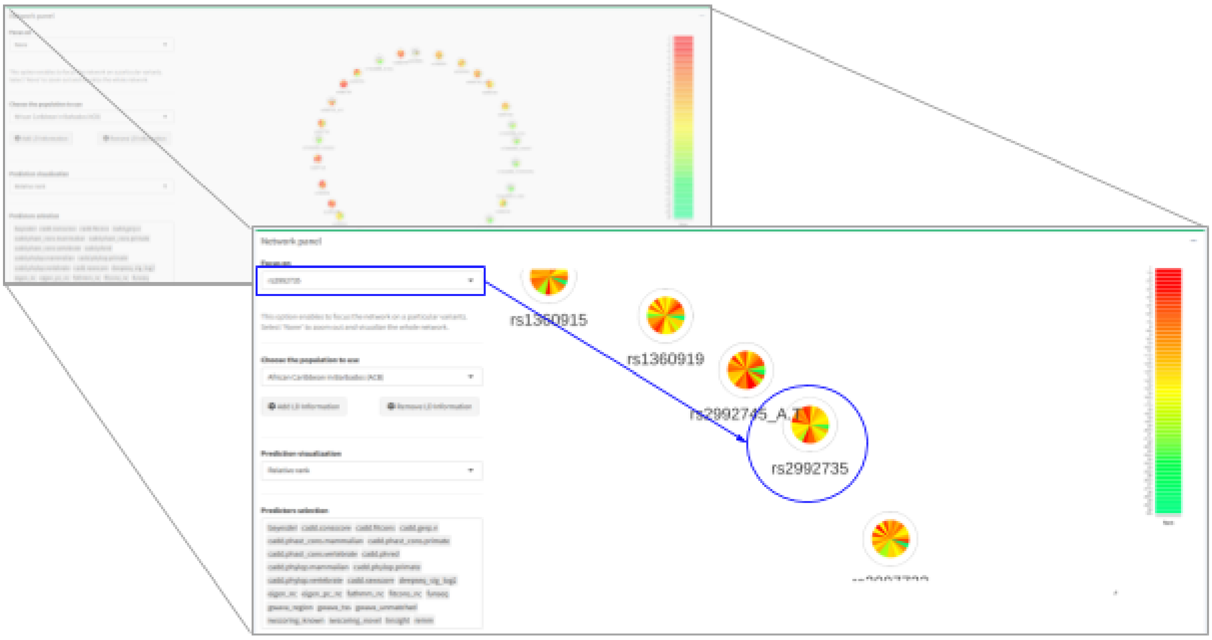

Linkage disequilibrium

By default, no linkage disequilibrium (LD) data are shown. To map LD on the network edges, choose a 1000 Genomes population and press "Add LD information" in the left panel.

This process can take a few seconds (up to 1 minute)

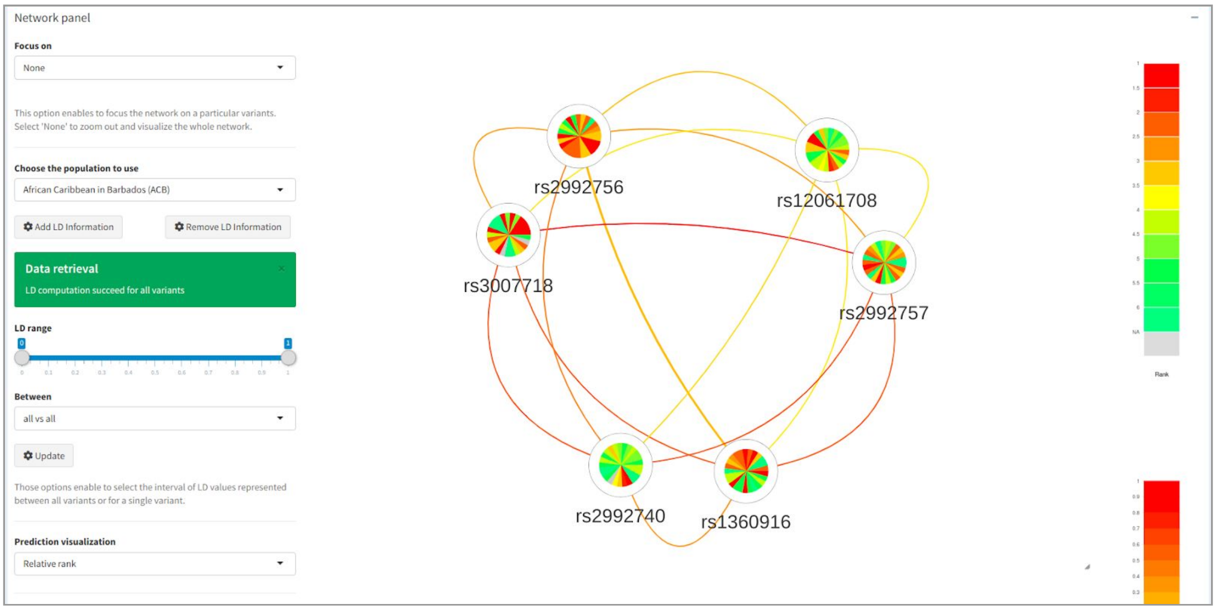

You can restrict the LD range you want to display after doing this, press "Update" to update the network.

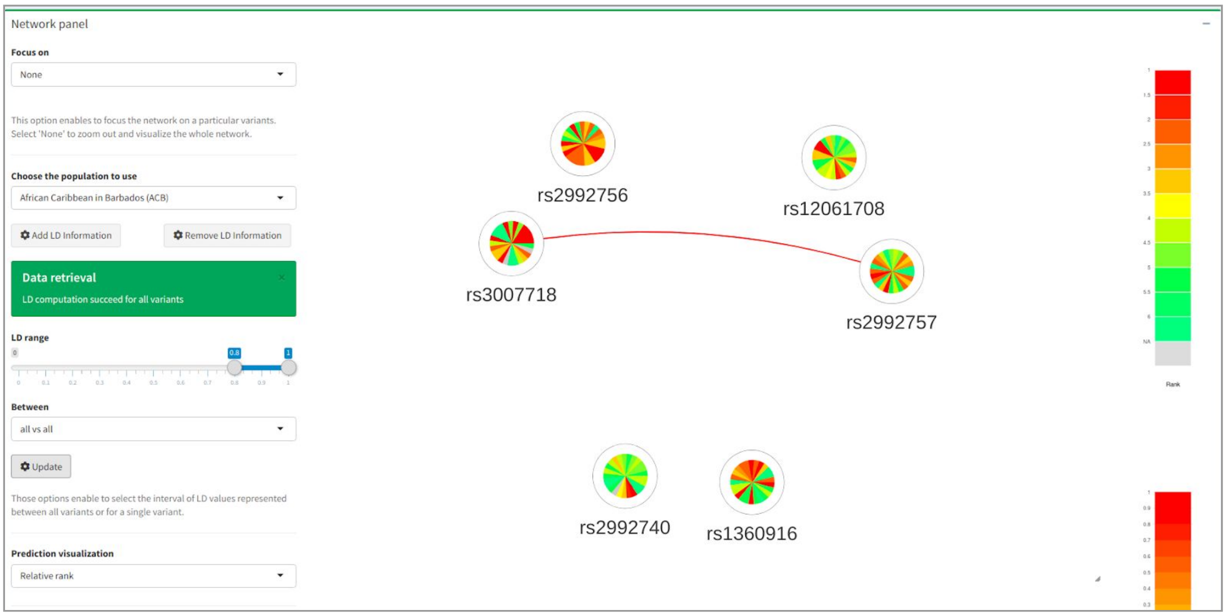

You can use the "Between" option to restrict the LD information to a particular variant. Press "Update" to update the visualization.

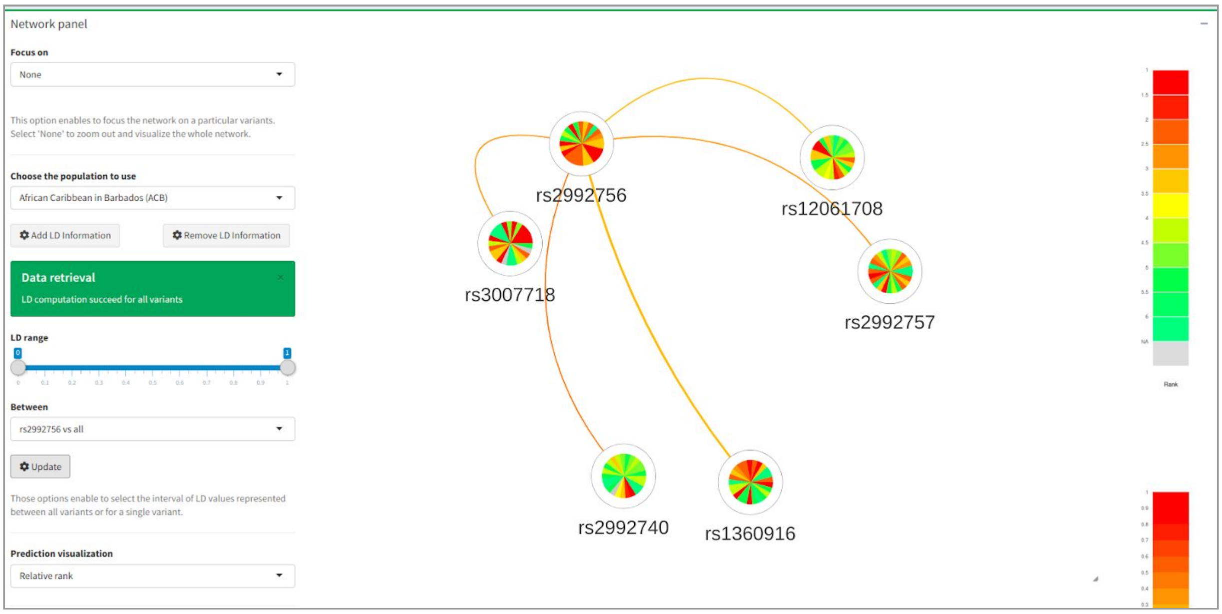

These two parameters can be combined to display a specific range of LD for a particular variant. This is very useful when dealing with big networks.

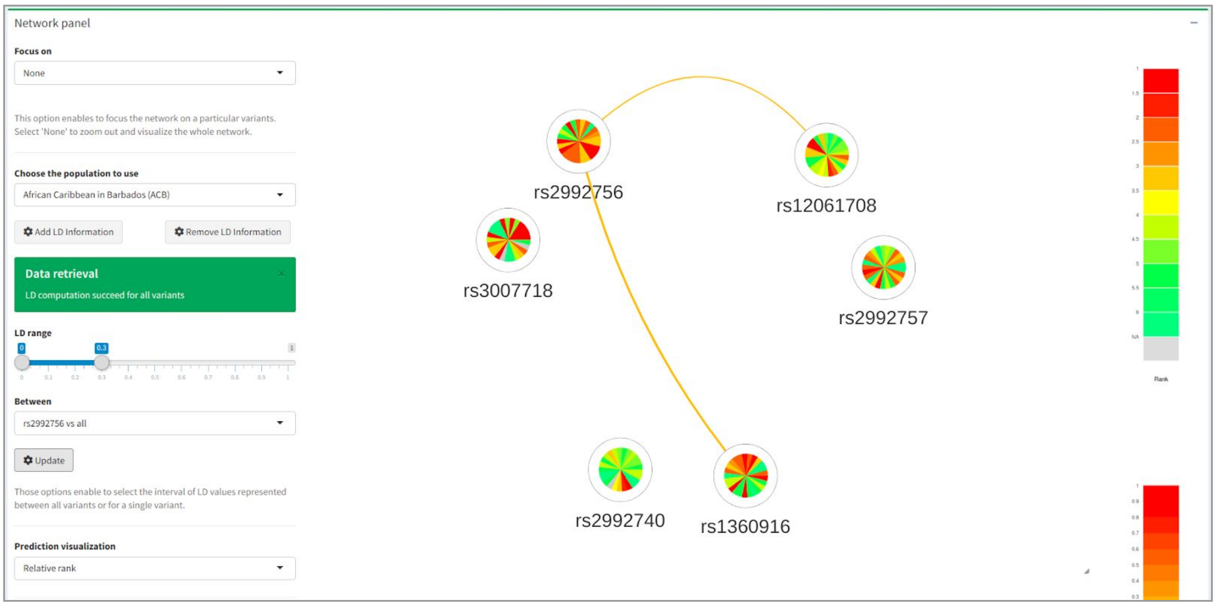

Score visualization

You can change the visualization through the dropdown list "Prediction visualisation". For example, the intra-predictor ranks can be ordered by color to emphasize the rank distribution.

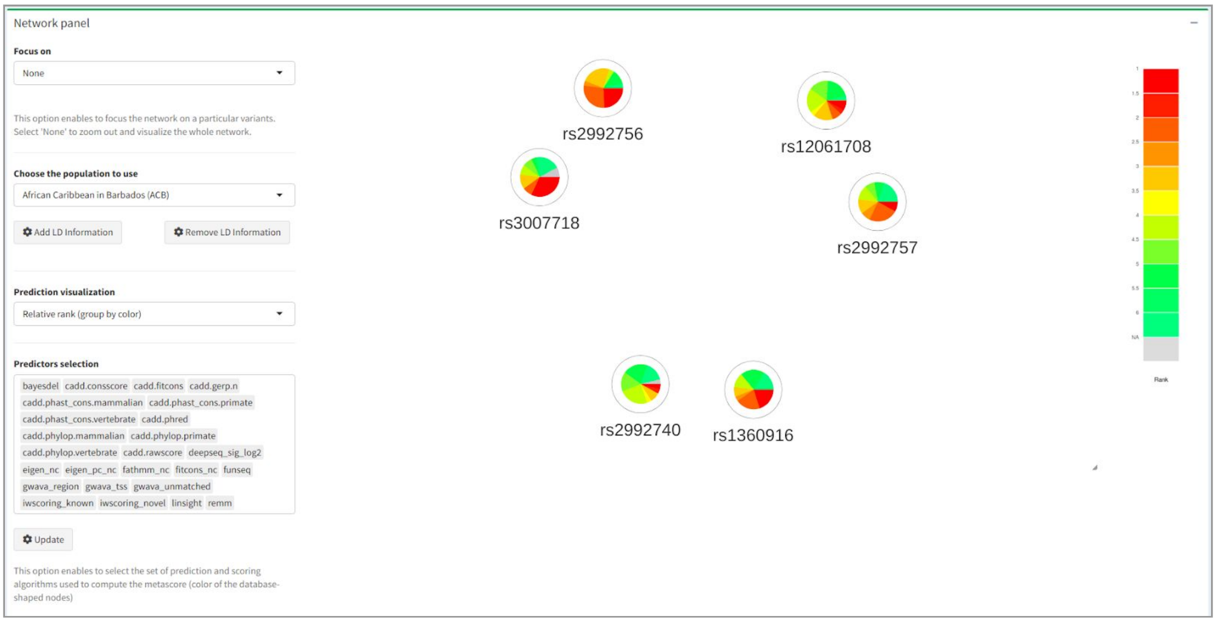

You can choose to visualize the absolute scores ordered by predictors or by color gradient.

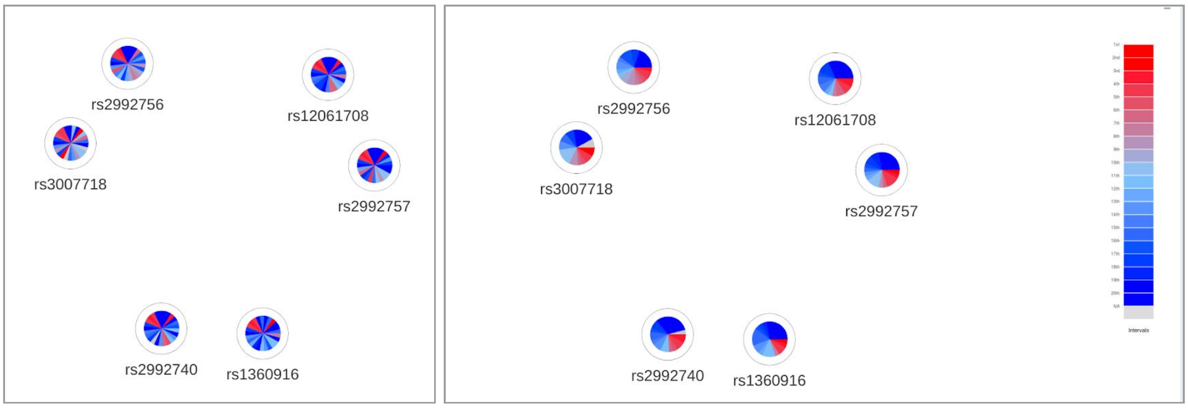

The dropdown list enables to select a global mean rank according to the substitution method of your choice among “mean”, and “median”.

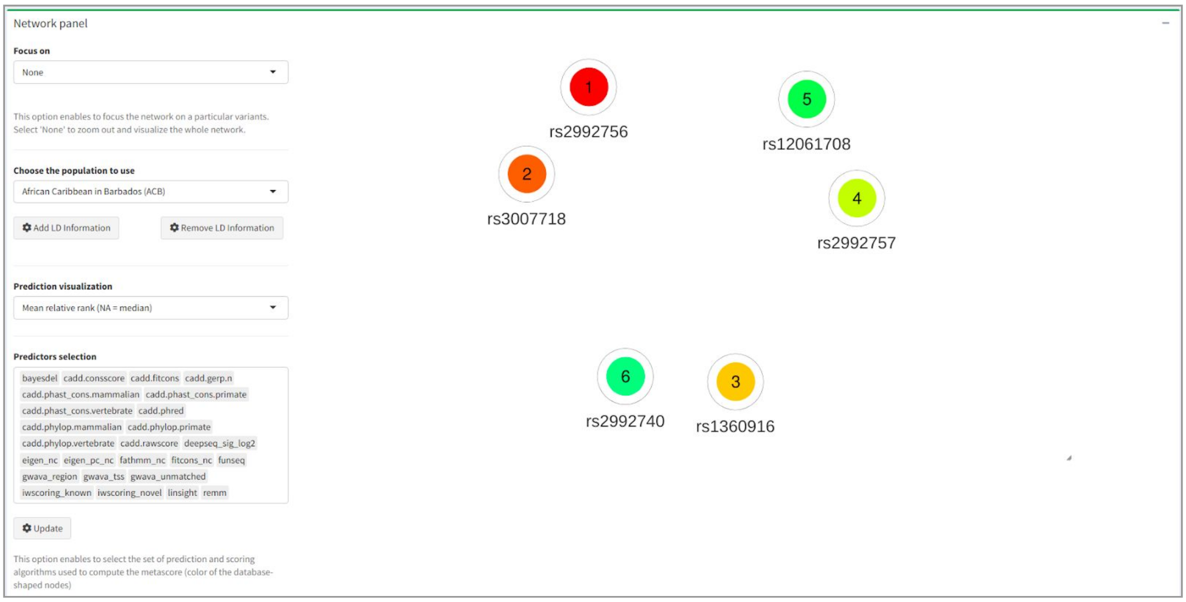

Predictors selection

Predictors providing predictions for at least one variant in the selection are displayed in the “Prediction selection” area. You can easily select a subset of predictors you are interested in by adjusting the list of predictors.

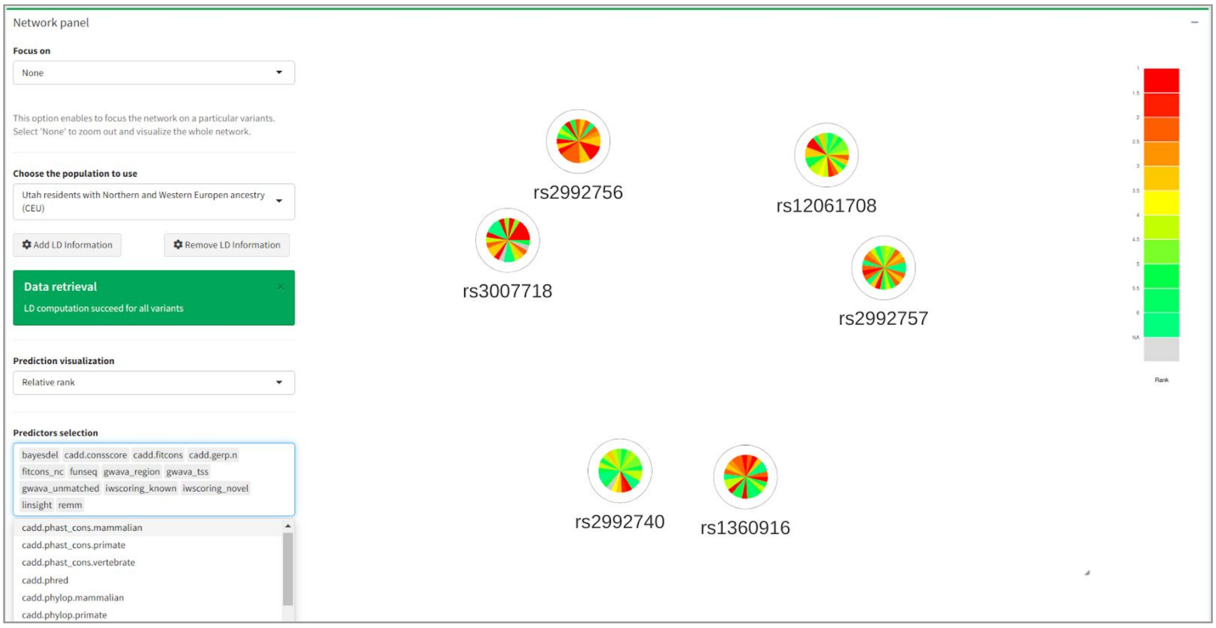

After doing so press “Update” to update the network.

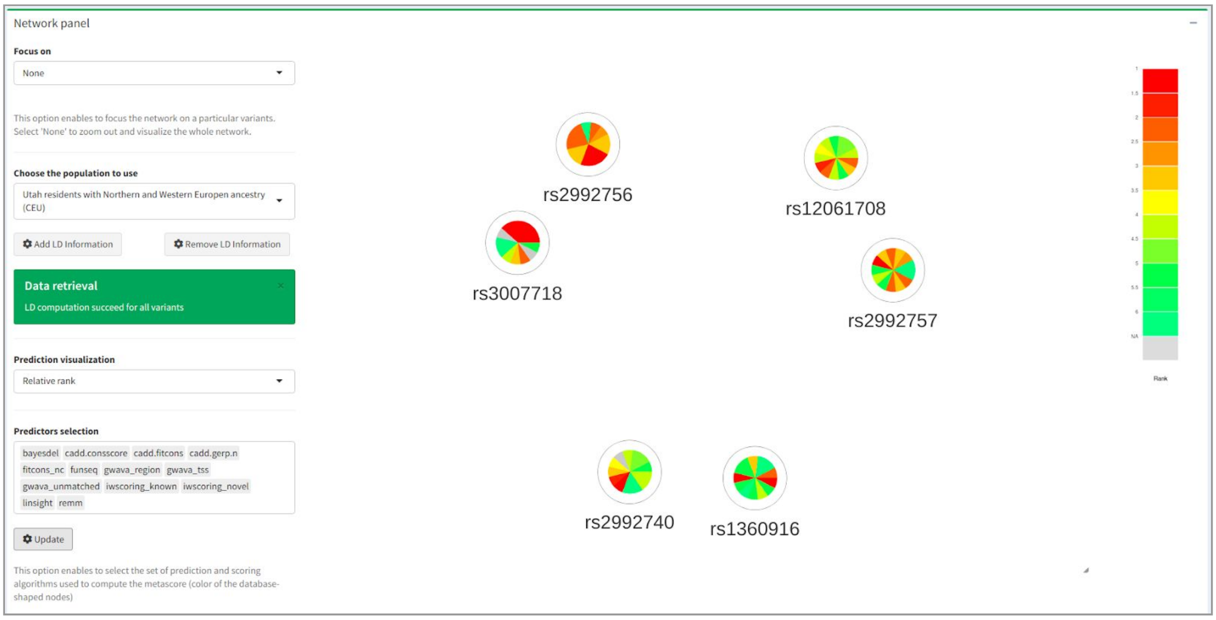

Complementary information

The predictor descriptions are available in the sidebar application.

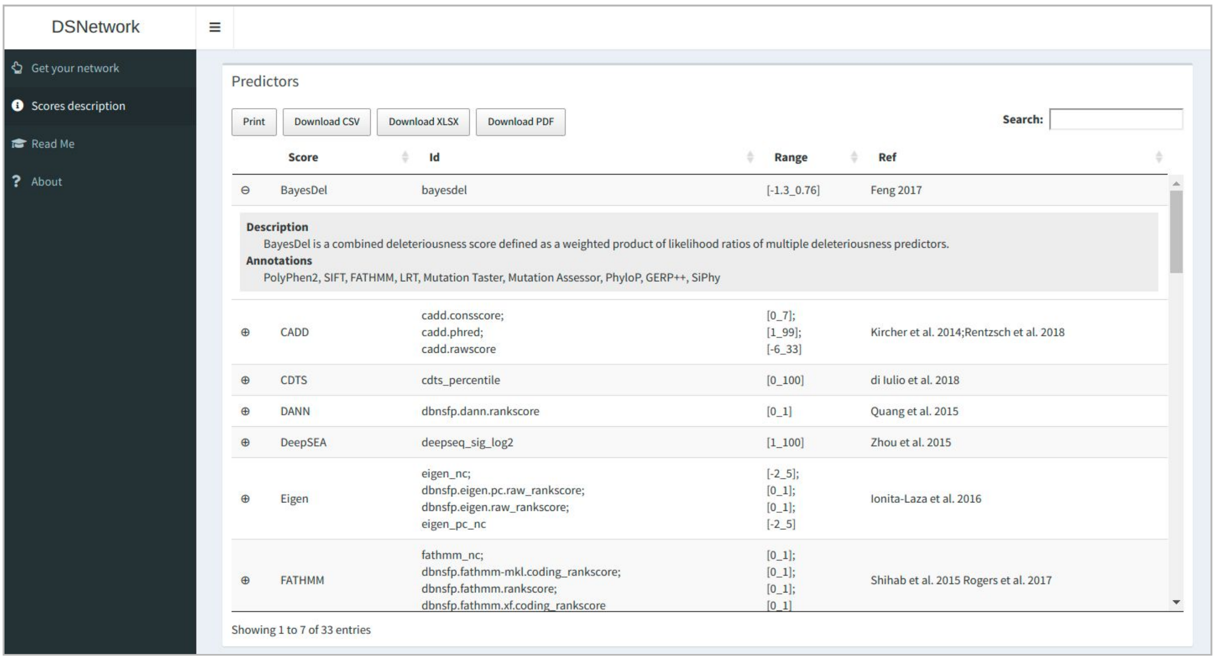
DSNetwork
Get your network
Scores description
Read Me
About
Predictors
Print
Download CSV
Download XLSX
Download PDF
Search:
Score
Id
Range
Ref
BayesDel
bayesdel
[-1.3_0.76]
Feng 2017
Description
BayesDel is a combined deleteriousness score defined as a weighted product of likelihood ratios of multiple deleteriousness predictors.
Annotations
PolyPhen2, SIFT, FATHMM, LRT, Mutation Taster, Mutation Assessor, PhyloP, GERP++, SiPhy
CADD
cadd.consscore;
cadd.phred;
cadd.rawscore
[0_7];
[1_99];
[-6_33]
Kircher et al. 2014;Rentzsch et al. 2018
CDTS
cdts_percentile
[0_100]
di Iulio et al. 2018
DANN
dbnsfp.dann.rankscore
[0_1]
Quang et al. 2015
DeepSEA
deepseq_sig_log2
[1_100]
Zhou et al. 2015
Eigen
eigen_nc;
dbnsfp.eigen.pc.raw_rankscore;
dbnsfp.eigen.raw_rankscore;
eigen_pc_nc
[-2_5];
[0_1];
[0_1];
[-2_5]
Ionita-Laza et al. 2016
FATHMM
fathmm_nc;
dbnsfp.fathmm-mkl.coding_rankscore;
dbnsfp.fathmm.rankscore;
dbnsfp.fathmm.xf.coding_rankscore
[0_1];
[0_1];
[0_1];
[0_1]
Shihab et al. 2015 Rogers et al. 2017
Showing 1 to 7 of 33 entries
